# Supplementary material for: Activation of mitochondrial TUFM ameliorates metabolic dysregulation through coordinating autophagy induction
Source: Commun Biol. 2021 Jan 4;4:1. doi: 10.1038/s42003-020-01566-0 (PMC7782552; doi:10.1038/s42003-020-01566-0)
Supplement: Supplementary file 2 — Supplementary Information [file 42003_2020_1566_MOESM2_ESM.pdf]

Supplementary Information

Activation of mitochondrial TUFM ameliorates metabolic dysregulation through coordinating autophagy induction

Dasol Kim[1, ¶], Hui-Yun Hwang[1, ¶], Eun Sun Ji[2], Jin Young Kim[2], Jong Shin Yoo[2], and Ho Jeong Kwon[1]*

[1]*Chemical Genomics Global Research Laboratory, Department of Biotechnology, College of Life Science and Biotechnology, Yonsei University, Seoul 03722, Republic of Korea.*

[2]*Biomedical Omics Group, Korea Basic Science Institute, Ochang, Chungbuk 28119, Republic of Korea*

¶These authors contributed equally to this work.

*Corresponding Author: Prof. Ho Jeong Kwon

E-mail: kwonhj@yonsei.ac.kr. Tel: +82-2-2123-5883

Supplementary Figure 1

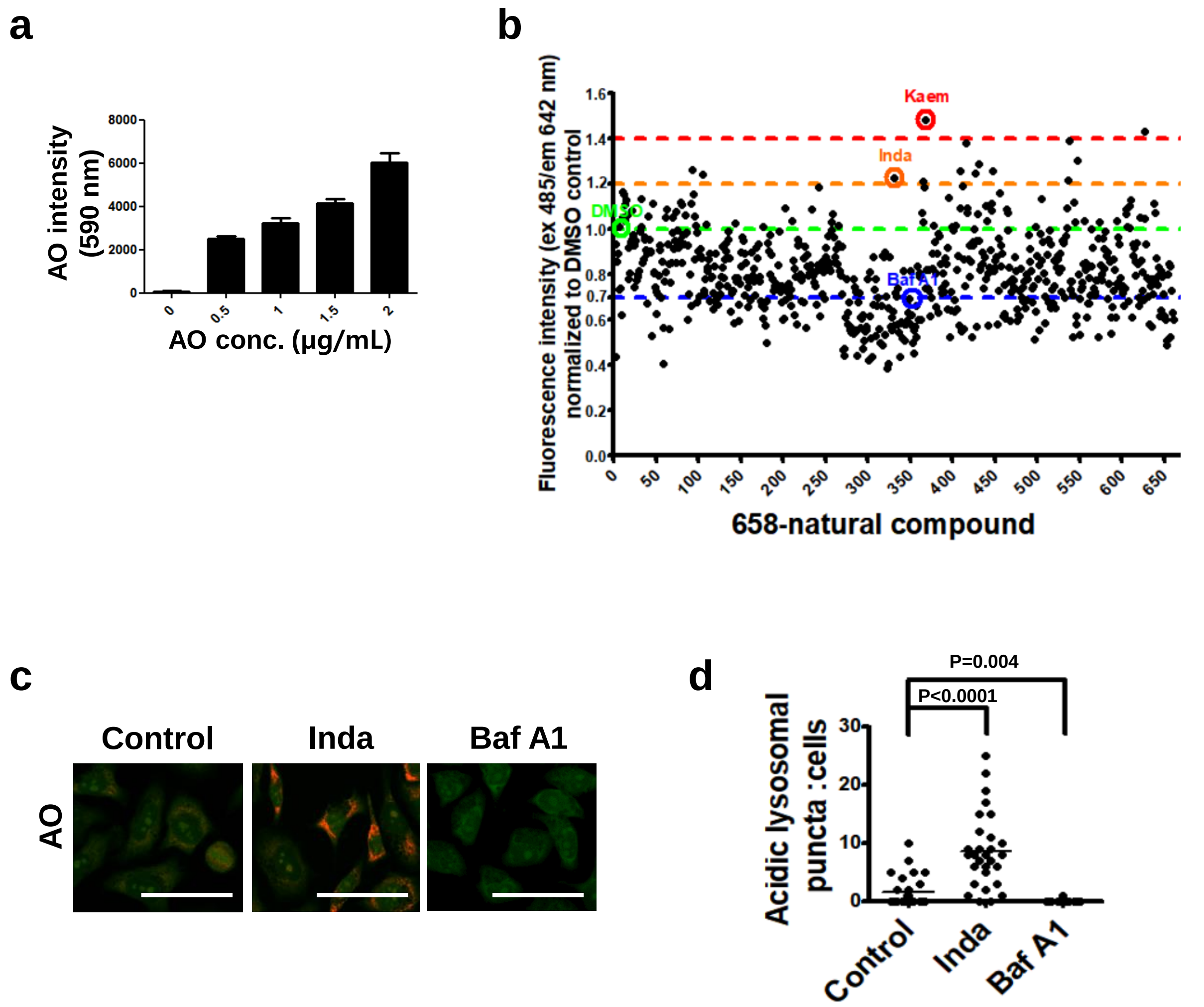

Supplementary Fig. 1 Kaempferide (Kaem) induces lysosomal activity in phenotypic screen. **a** The correlation of florescence intensity with dose of acridine orange (AO) dye was validated by the Victor3 plate reader (Perkin Elmer). **b** HeLa cells were treated with chemicals from a 658-natural compound library, indatraline (Inda, a positive control), and bafilomycin A1 (Baf A1, a negative control) for 24 h, then stained with acridine orange. Acridine orange (AO) intensity of each wells was normalized to DMSO control intensity. The green, orange, and blue dotted lines indicate AO intensity of DMSO control (1), and indatraline (Inda, 1.2 folds), and bafilomycin A1 (Baf A1, 0.7 folds) treated well, respectively. kaempferide (Kaem) treated intensity is indicated in red circle above red dotted line which indicates 1.4 folds increased intensity. Hit compound Kaem was used for experiments throughout this study. **c, d** DMSO control, indatraline, and bafilomycin A1 treated HeLa cells were stained with acridine orange (AO), and confocal microscopy was performed **c**. Graph shows mean ± SD (n=30) of acidic vesicles per cells **d**. Inda, 5 µM, Baf A1, 10 nM, Scale bar, 50 µm.

Supplementary Figure 2

a

Compound	MW	XLogP	H-bond acceptor	H-bond donor	Rotation al bonds
Kaempferide	300.26	2.2	6	3	2
Tiliroside	594.52	2.5	13	7	8
Asperosaponin VI	929.10	0.8	18	11	10
aurantio-obtusin	330.29	2.7	7	3	2
rebaudioside A	967.01	-2.8	23	14	13

b

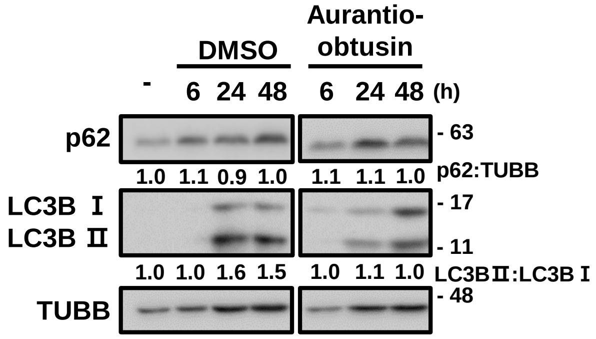

Supplementary Fig. 2 Hit selection from screen. **a** Chemical information indicating whether each of them satisfies Lipinski's 5-rule. Each numerical information was from pubchem (https://pubchem.ncbi.nlm.nih.gov/). MW, molecular weight. **b** HeLa cells were treated with DMSO control or Aurantio-obtusin for indicated period. Cell extracts were subjected to western blot analysis using antibodies for LC3B and p62. Representative images with the numbers below for intensity of p62 and LC3B-II immunoblot bands normalized to TUBB and LC3B-Ⅰ, respectively. The blots were processed in parallel. Aurantio-obtusin, 10 μM.

Supplementary Figure 3

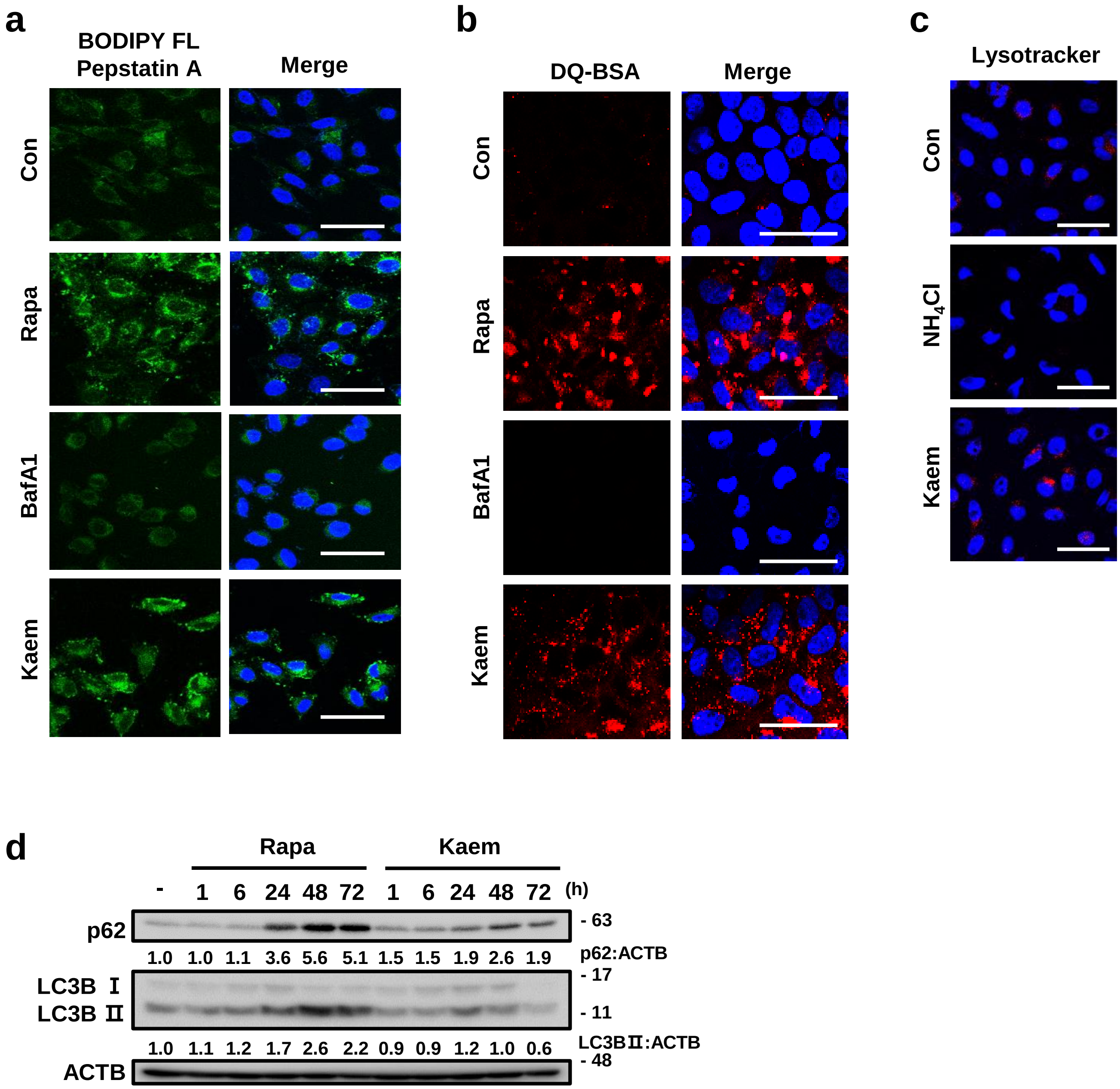

Supplementary Fig. 3 Kaem induces autophagy flux in HeLa cells. **a** HeLa cells were treated with DMSO control, rapamycin (Rapa), bafilomycin A1 (Baf A1), and Kaem for 6 h, respectively and stained with Pepstatin-BODIPY. Confocal microscopy was performed. Kaem, 20 µM, Rapa, 10 µM, Baf A1, 10 nM, Scale bar, 50 µm. **b** HeLa cells were treated with DMSO control, rapamycin (Rapa), bafilomycin A1 (Baf A1), and Kaem for 6 h, respectively, and treated with DQ-BSA. Confocal microscopy was performed. Kaem, 20 µM, Rapa, 10 µM, Baf A1, 10 nM, Scale bar, 50 µm. **c** HeLa cells were treated with DMSO control, NH_4Cl, and Kaem for 24 h, respectively, and stained with Lysotracker. Confocal microscopy was performed. Kaem, 20 µM, NH_4Cl, 10 mM, Scale bar, 50 µm. **d** HeLa cells were treated with rapamycin (Rapa) or Kaem for indicated period. Cell extracts were subjected to western blot analysis using antibodies for LC3B and p62. Numbers below p62 and LC3-II immunoblot bands indicates fold changes normalized to ACTB. Kaem, 20 µM, Rapa, 10 µM.

Supplementary Figure 4

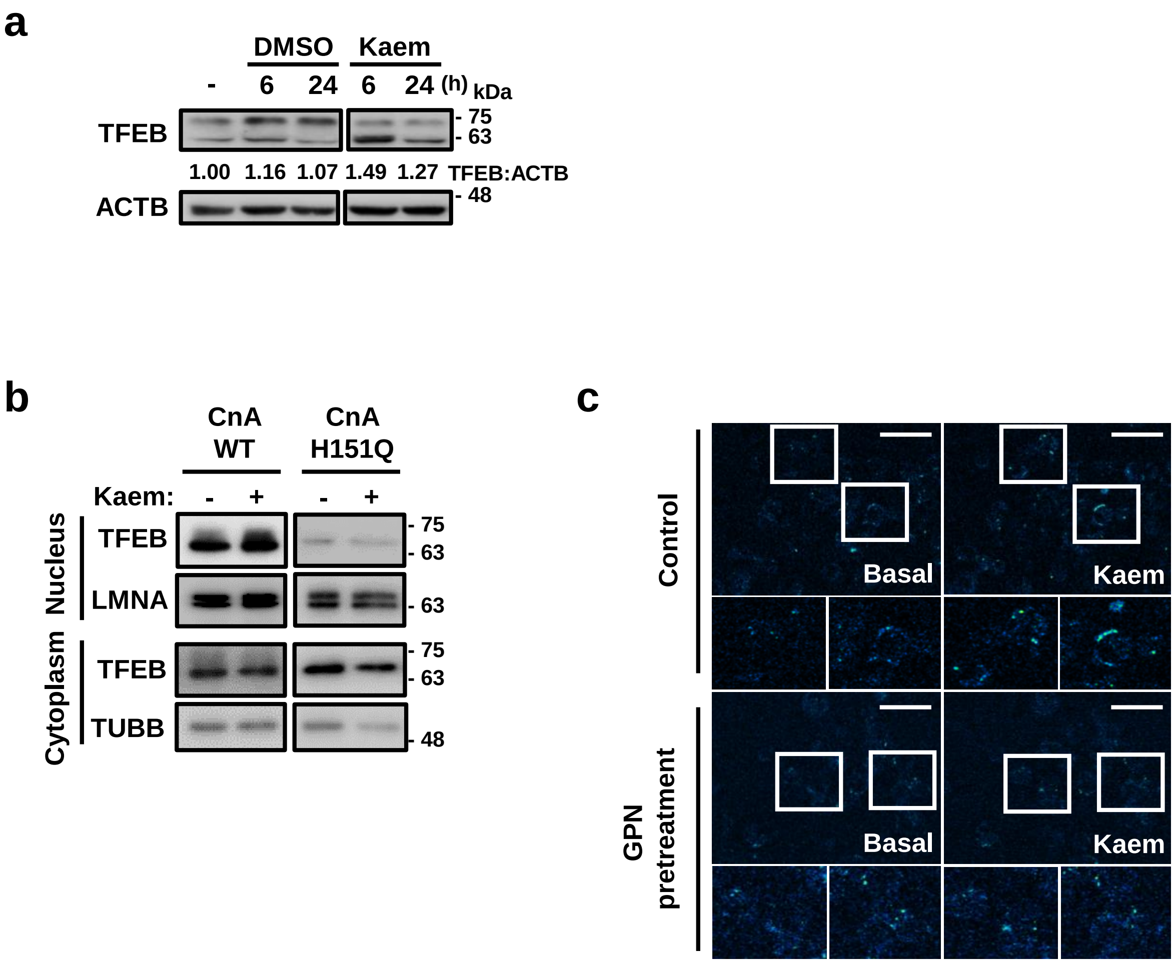

Supplementary Fig. 4 Kaem induces lysosomal Ca^{2+} to activate TFEB. **a** HeLa cells were treated with DMSO control or Kaem for 6, 24 h. Cell extracts were subjected to western blot analysis using antibody for TFEB. The shifted immunoblot bands of TFEB indicate phosphorylated state (upper) or not (below). Numbers below TFEB immunoblot bands indicates fold changes normalized to ACTB. The blots were processed in parallel. Kaem, 20 μM. **b** HeLa cells were transfected with or without HA-ΔCnA-H151Q construct, and treated with Kaem for 6 h, fractionated, and immunoblotted using antibody for TFEB. The blots were processed in parallel. Kaem, 20 μM. WT, wild-type. **c** HeLa cells were transfected with GCaMP3-ML1 encoding a lysosome-specific Ca^{2+} probe and then treated with DMSO control (Basal) or Kaem, with or without glycyl-L-phenylalanine-β-naphthylamide (GPN) pretreatment for 1 h. Lysosomal Ca^{2+} release was visualized by confocal microscopy. Kaem, 20 μM, GPN, 100 μM, Scale bar, 20 μm.

Supplementary Figure 5

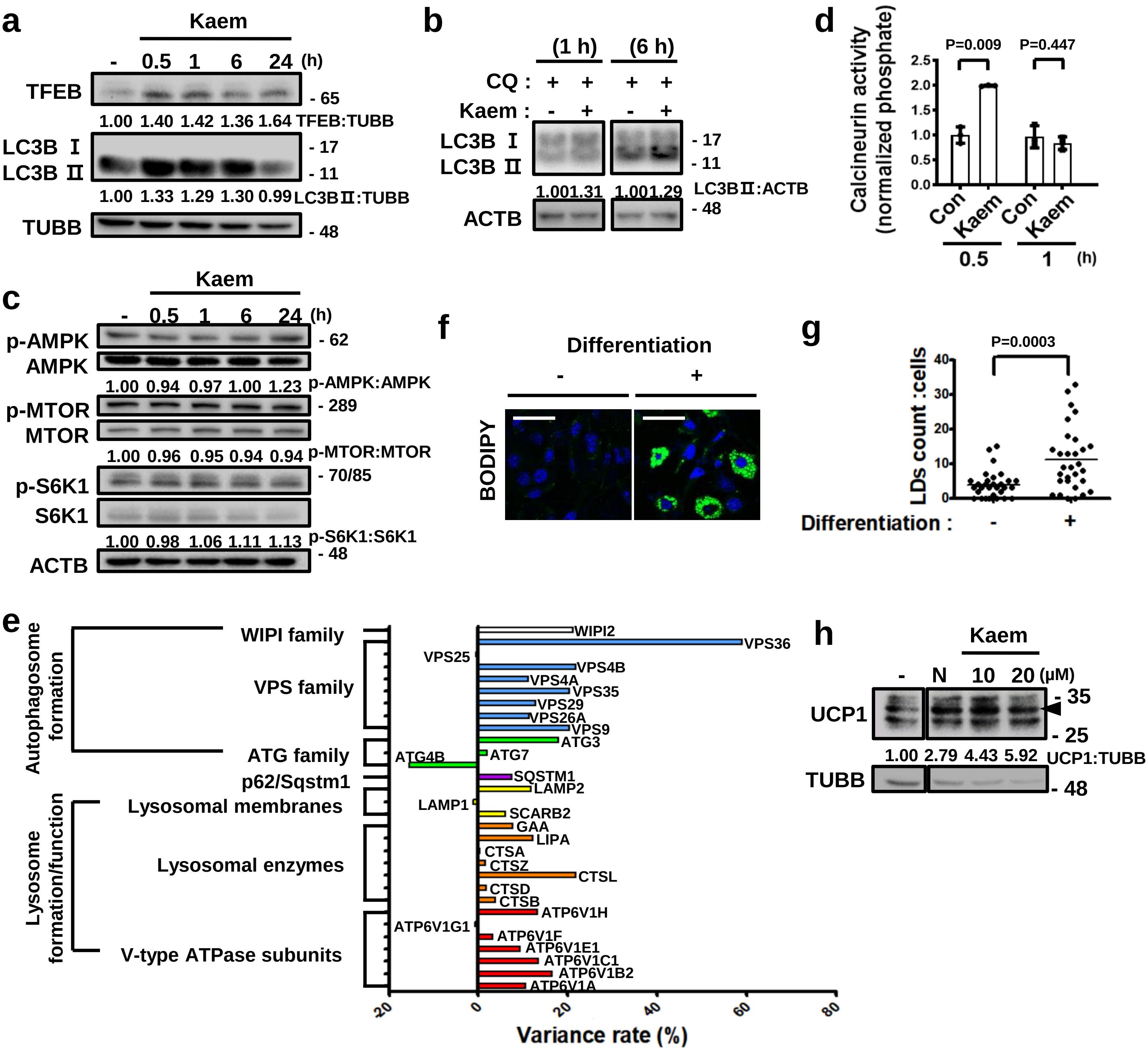

Supplementary Fig. 5 Kaem induces autophagy through TFEB activation without MTOR-AMPK perturbation in 3T3-L1 adipocytes. **a** 3T3-L1 cells were treated with Kaem in time course for indicated period. Cell extracts were subjected to western blot analysis using antibodies for TFEB and LC3B. Numbers below TFEB and LC3BⅡ immunoblot bands indicates fold changes normalized to TUBB. Kaem, 20 μM. **b** 3T3-L1 cells were treated with Kaem in the presence or absence of CQ for 1, 6 h. Cell extract were subjected to western blot analysis using antibody for LC3B. Numbers below LC3B-Ⅱ immunoblot bands indicates fold changes normalized to ACTB. The blots were processed in parallel. Kaem, 20 μM, CQ, 10 μM. **c** 3T3-L1 cells were treated with Kaem for indicated period. Cell extract was subjected to western blot analysis using antibodies for p-AMPK, AMPK, p-MTOR, MTOR, p-S6K1, S6K1. Numbers below each phosphorylated proteins immunoblot bands indicates fold changes normalized to unphosphorylated. Kaem, 20 μM. **d** 3T3-L1 cells were treated with Kaem for 0.5, 1 h. Calcineurin activity assay was conducted according to the manufacture's direction. Absorbance ratio was measured at 620 nm. Kaem, 20 μM. Graphs represent mean ±SD from three independent experiments. **e** 3T3-L1 cells were treated with Kaem for 24 h. The cytoplasmic proteins were subjected to LC-MS/MS analysis after TMT-labeled. The graph for autophagy related proteins level was shown (n=1). **f, g**) 3T3-L1 cells were differentiated for 5 days and confocal microscopy was performed after BODIPY 494/503 staining. Representative images **f** and graph for the number of lipid droplets (LDs) **g**. Graph shows mean ± SD (n=30). Scale bar, 50 μm. **h** 3T3-L1 cells were treated with norepinephrine (NE) or Kaem for 24 h. Cell extracts were subjected to western blot analysis using antibody for UCP1. Numbers below UCP1 immunoblot bands indicates fold changes normalized to TUBB. The blots were processed in parallel. NE, 10 μM. Statistical significance was assessed by student's *t*-test.

Supplementary Figure 6

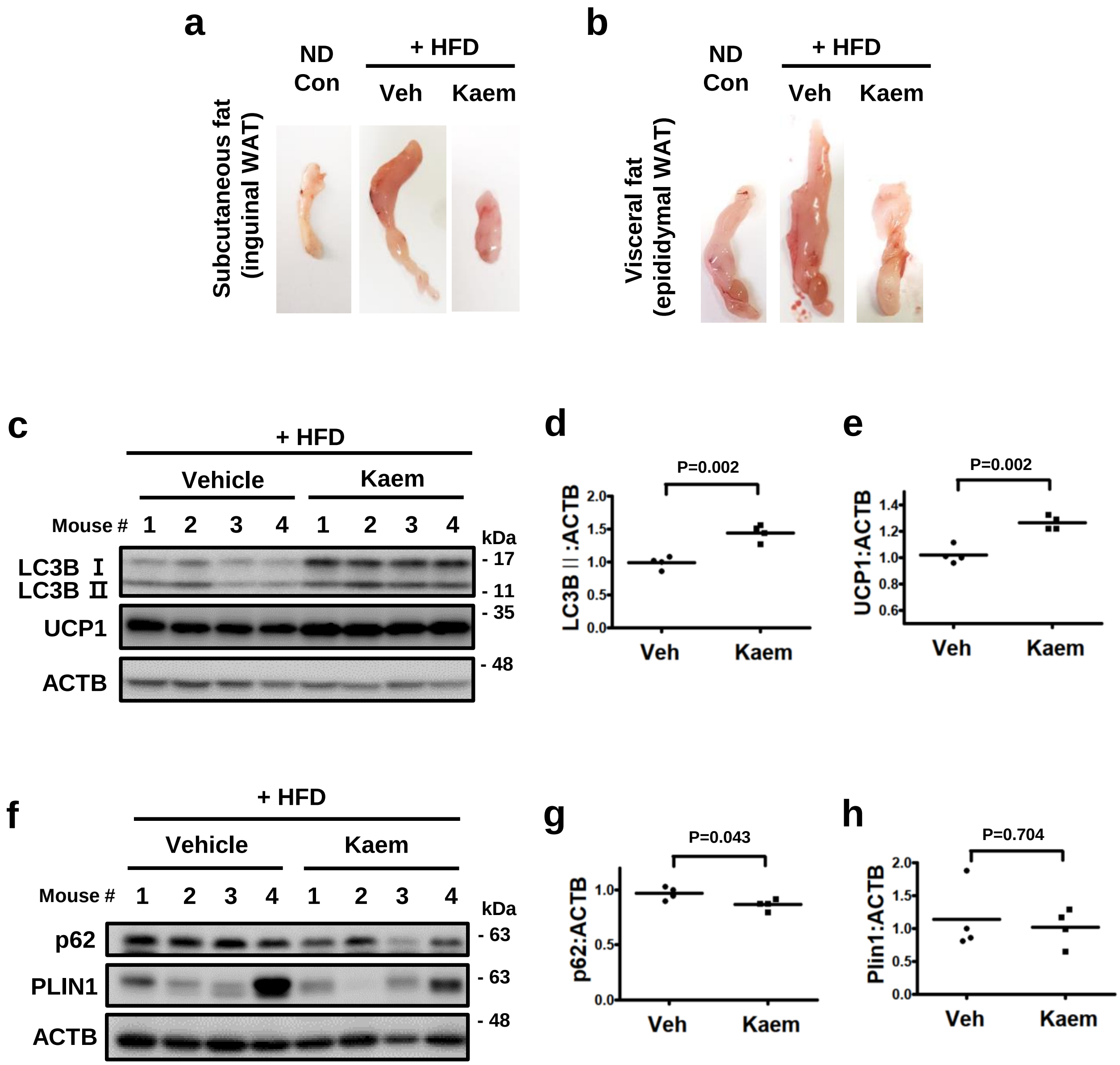

Supplementary Fig. 6 Kaem ameliorated fat deposition in high-fat diet (HFD)-induced obese mice with enhancing autophagy *in vivo*. **a, b** C57BL/6j mice were fed with HFD for 2 months, vehicle or Kaem were treated on every 2 days for 2 months respectively (n=4). Intraperitoneal injection, Kaem, 10 mg/kg. After sacrifice, subcutaneous **a** and visceral **b** fat tissues of mice were obtained. Representative photo images were shown. ND, normal diet. **c-e** Each mice interscapular brown adipose tissues (BATs) lysate was subjected to western blot analysis using antibodies for LC3B and UCP1. Representative images **c** and the graph intensity of LC3B **d** and UCP1 **e** immunoblot bands normalized to ACTB. Graphs represent mean ±SD (n=4). **f-h** Each mice visceral white adipose tissues (WATs) lysate was subjected to western blot analysis using antibodies for p62 and PLIN1. Representative images **f** and the graph intensity of p62 **g** and Plin1 **h** immunoblot bands normalized to ACTB. Graphs represent mean ±SD (n=4). Statistical significance was assessed by student's t-test. ***P<0.001; **P<0.01; *P<0.05.

Supplementary Figure 7

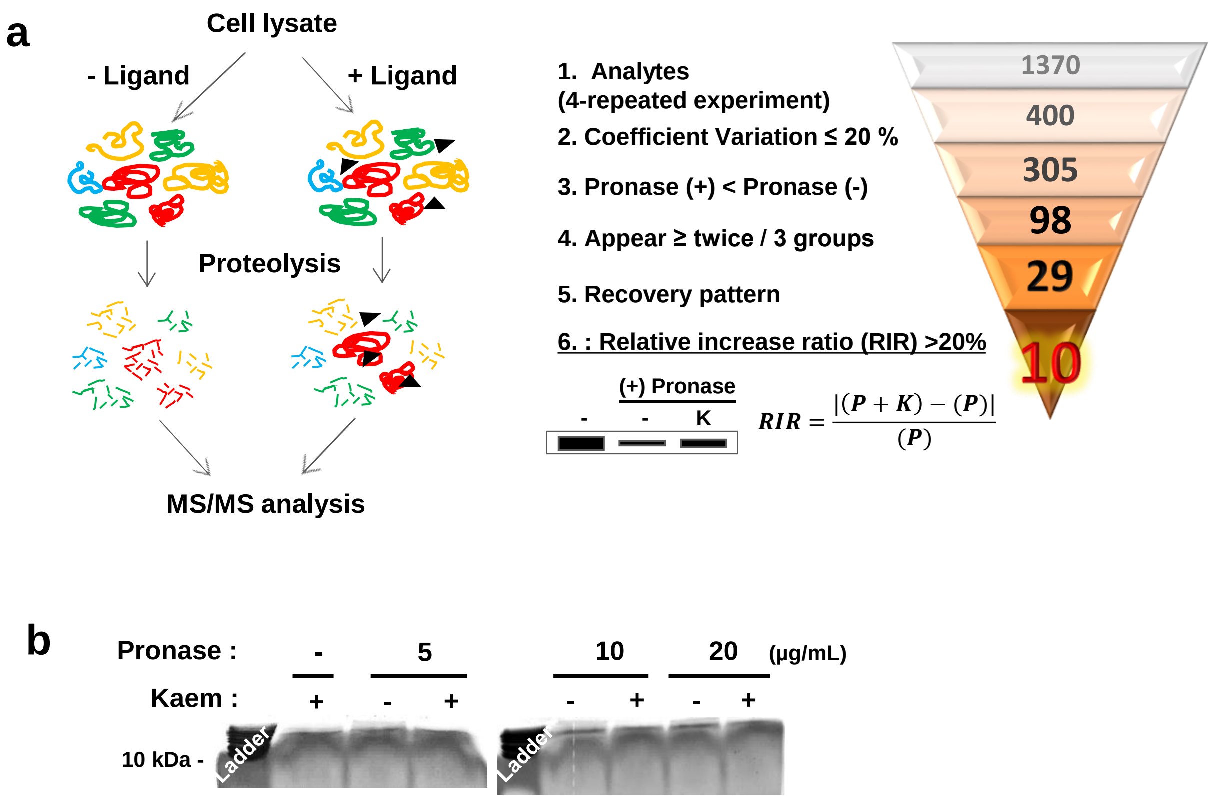

Supplementary Fig. 7 Target protein identification of Kaem. **a** Schematic figure of DARTS experimental process (left), and steps for selection of target candidates (right). **b** 3T3-L1 cell lysate was treated with pronase for 10 minutes with or without Kaem pre-treatment, and subjected to SDS-PAGE shortly. Total proteins in the gels were stained with Coomassie brilliant blue (CBB) stain to discern proteome in size over 10 kDa (mol-cut).

Supplementary Figure 8

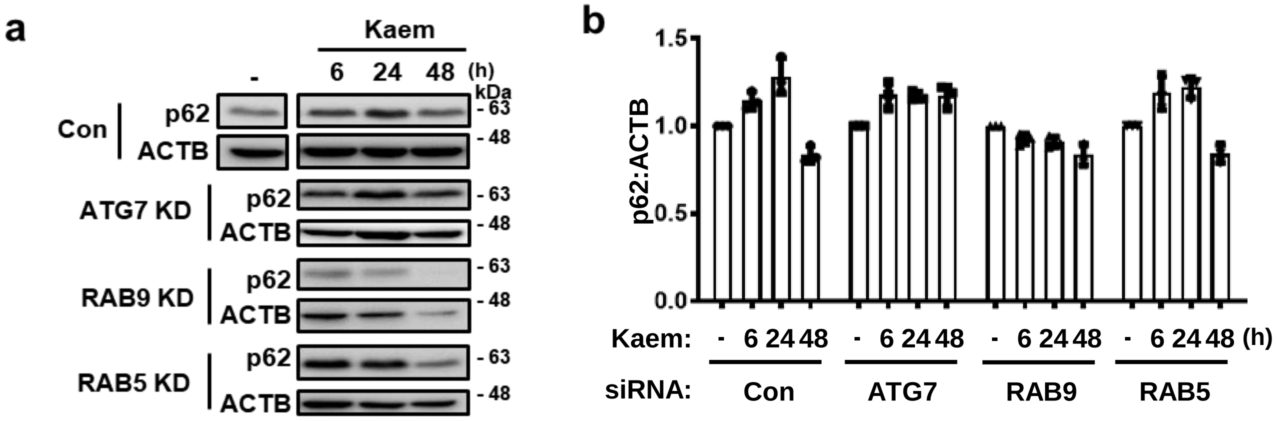

Supplementary Fig. 8 Kaem requires ATG7 to induce autophagy turn-over. **a** HeLa cells were transfected with or without siRNA targeting ATG7, RAB9, and RAB5 respectively for 24 h, followed by Kaem treatment for 6, 24, and 48 h. Cell extract was subjected to western blot analysis using indicated antibodies. Representative images **a** and the graph for intensity of p62 immunoblot bands normalized to ACTB **b**. The blots were processed in parallel. Kaem, 20 μM. Graph shows mean ± SD from three independent experiments.

Supplementary Figure 9

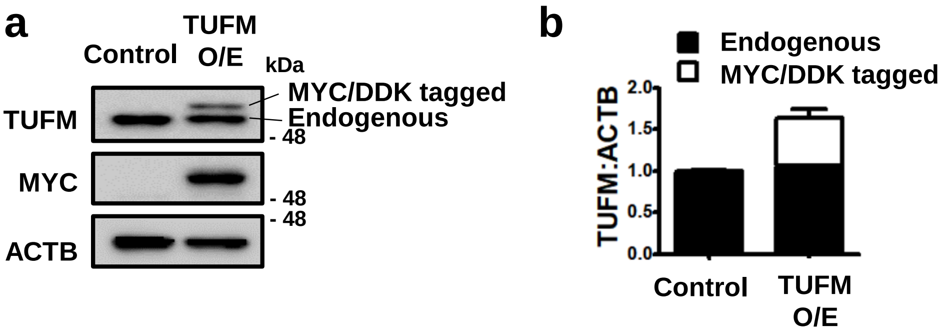

Supplementary Fig. 9 Regulation of target proteins in expression levels. **a, b** Huh7 cells were transfected with TUFM construct (MYC/DDK tagged) for 24 h. Cell extract was subjected to western blot analysis using antibodies for TUFM and MYC. Representative images **a** and the graph for intensity of TUFM and MYC tagged TUFM immunoblot bands normalized to ACTB **b**. Graphs represent mean ±SD from three independent experiments.

Supplementary Figure 10

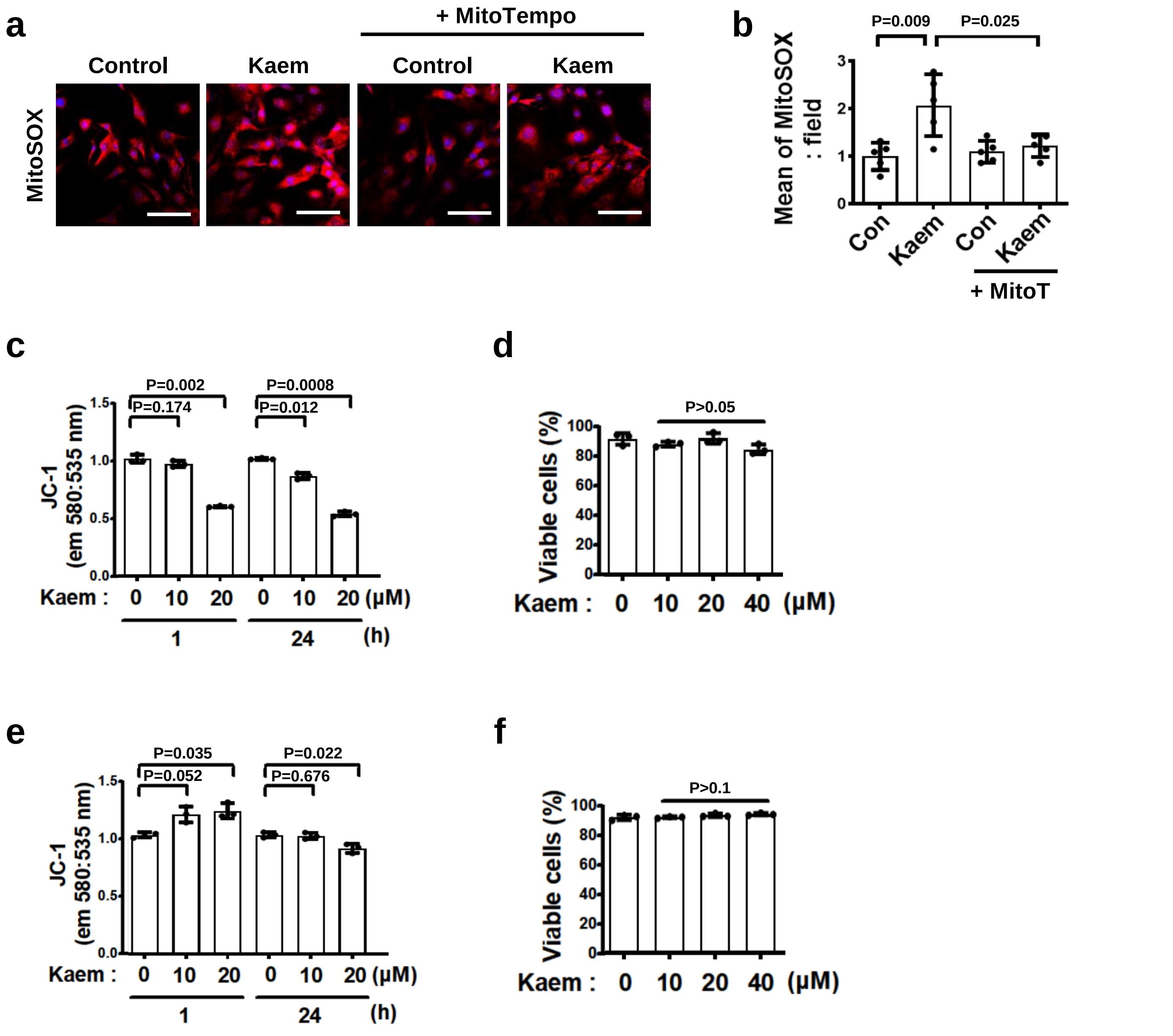

Supplementary Fig. 10 Kaem slightly enhances mitochondrial ROS production without severe toxicity to the cells. **a, b** 3T3-L1 cells were treated with Kaem in the presence or absence of mitoTempo (MitoT) for 24 h. Cells were stained with mitoSOX, and fluorescence microscopy was performed. Representative images **a** and the graph for fluorescence intensity measured using ImageJ 2 **b**. Kaem, 20 µM, mitoTempo, 100 µM. Scale bar, 100 µm. Graphs represent mean ±SD from three different fields. **c** HeLa cells were treated with Kaem for indicated period, stained with JC-1. Fluorescence were measured by Victor plate reader. Graphs represent mean ±SD (n=3). **d** HeLa cells were treated with Kaem for 48 h, stained with trypan blue. Dye-positive (dead) and negative (viable) cells were counted. Graphs represent mean ±SD (n=3). **e** 3T3-L1 differentiated cells were treated with Kaem for indicated period, stained with JC-1. Fluorescence were measured by Victor plate reader. Graphs represent mean ±SD (n=3). **f** 3T3-L1 differentiated cells were treated with Kaem for 48 h, stained with trypan blue. Dye-positive (dead) and negative (viable) cells were counted. Graphs represent mean ±SD (n=3). Statistical significance was assessed by student's t-test. ****P*<0.001; ***P*<0.01; **P*<0.05.

Supplementary Figure 11

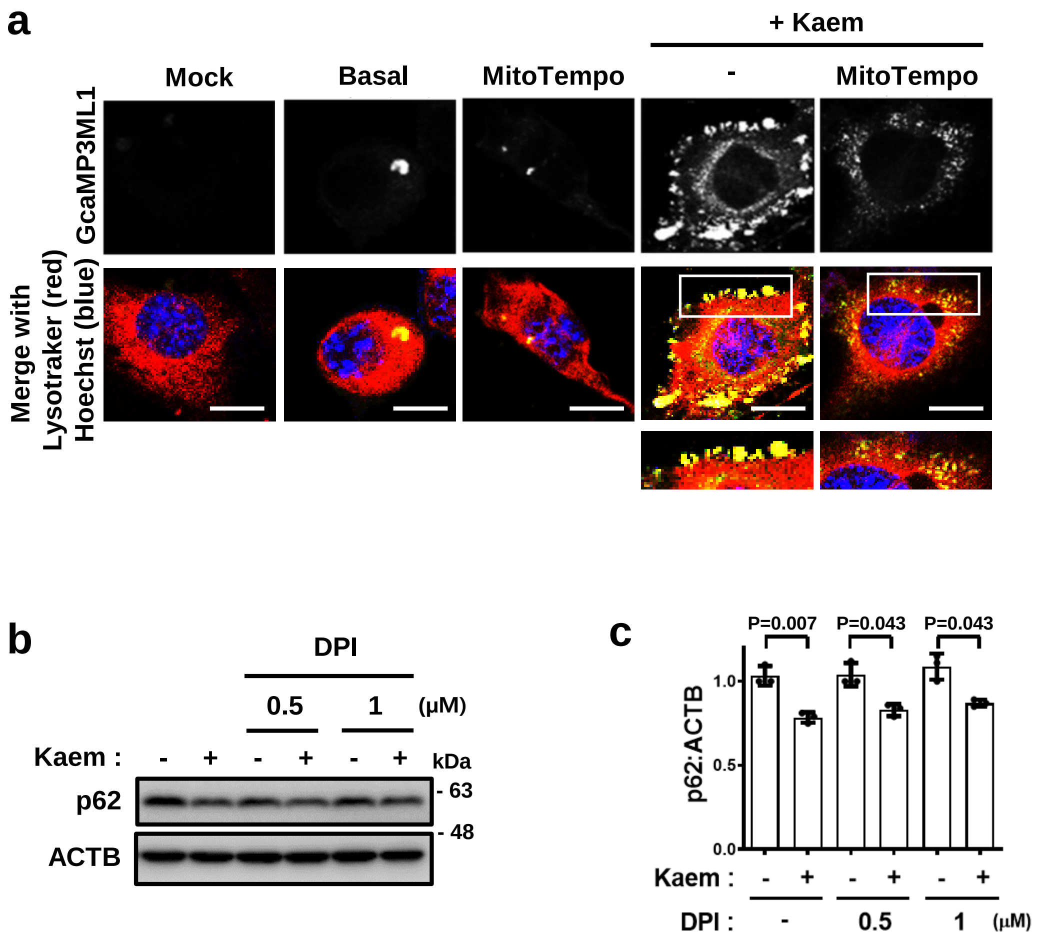

Supplementary Fig. 11 Kaem-induced Ca^{2+} release and autophagy is dependent on mtROS but not on cytoplasmic NOX. **a** 3T3-L1 cells were transfected with GCaMP3-ML1 encoding a lysosome-specific Ca^{2+} probe, and treated with Kaem with or without mitoTempo. Lysosomal Ca^{2+} release was visualized by confocal microscopy. Scale bar, 10 μm. **b, c** 3T3-L1 cells were treated with Kaem in presence or absence of diphenylene iodonium (DPI). Cell extract was subjected to western blot analysis using antibody for p62. Representative images **b** and the graph for intensity of p62 immunoblot bands normalized to ACTB **c**. Kaem, 20 μM, DPI, 0.5, 1 μM. Graphs represent mean ±SD from three independent experiments. Statistical significance was assessed by student's t-test. ****P*<0.001; ***P*<0.01; **P*<0.05.

Supplementary Figure 12

Protein	Variance rate ((Kaem-Control)/Control)(%)	
NDUFS8	23.1	Complex Ⅰ
NDUFS5	15.3	
NDUFB8	10.8	
NDUFA4	6.10	
NDUFA7,	0.44	
NDUFAF2	-5.43	
NDUFV2	-17.4	
SDHB	0.60	Complex Ⅱ
UQCRC1	16.0	Complex Ⅲ
UQCRH	9.83	
UQCRB	0.51	
UQCRFS1	-3.27	
COX5A	28.1	Complex Ⅳ
COX4I1	17.2	
COX5B	16.9	
COX6B1	4.01	
COX6C	0.23	
ATP5O	57.5	Complex Ⅴ
ATP5H	19.7	
ATP5A1	18.5	
ATP5B	2.07	
CLPP	37.5	Non-ETC proteins
HSPE1	33.3	
MRPL12	32.7	
IDH3A	29.1	
SSBP1	25.2	
MRPL39	23.4	
CYCS	22.5	
MRPS36	21.3	
GLRX5	20.0	
PDHA1	19.8	
SUCLG1	18.1	
MRPS6	17.6	
MRPL49	14.7	
PRDX5	13.2	
DLD	12.6	
HSPD1	11.9	
CYB5A	9.70	
VDAC1	7.93	
MDH2	6.48	
GLS	5.36	
TUFM	5.07	
VDAC2	4.44	
ETFA	-3.44	
ETFB	-3.72	
HSPA9	-3.73	
IDH2	-4.30	
GRPEL1	-4.48	
MRPL58	-5.18	
CYB5B	-36.5	

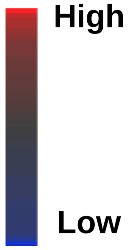

Supplementary Fig. 12 Kaem modulates expression level of mitochondrial ETC proteins. 3T3-L1 cells were treated with Kaem for 24 h. Mitochondrial proteins were fractionated and subjected to LC-MS/MS analysis after TMT-labeled. The heat map for mitochondrial ETC proteins level was shown.

Supplementary Figure 13

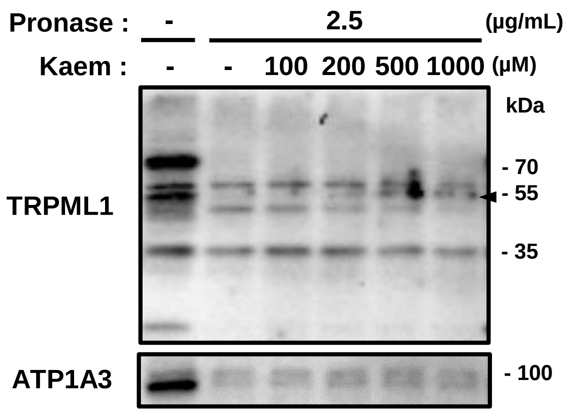

Supplementary Fig. 13 High dose of Kaem physically binds to TRPML1. 3T3-L1 cell lysate was treated with pronase for 10 minutes with or without Kaem pre-treatment in dose dependent manner (0, 100, 200, 500, and 1000 µM). Western blot analysis was subjected using indicated antibodies.

Supplementary Figure 14

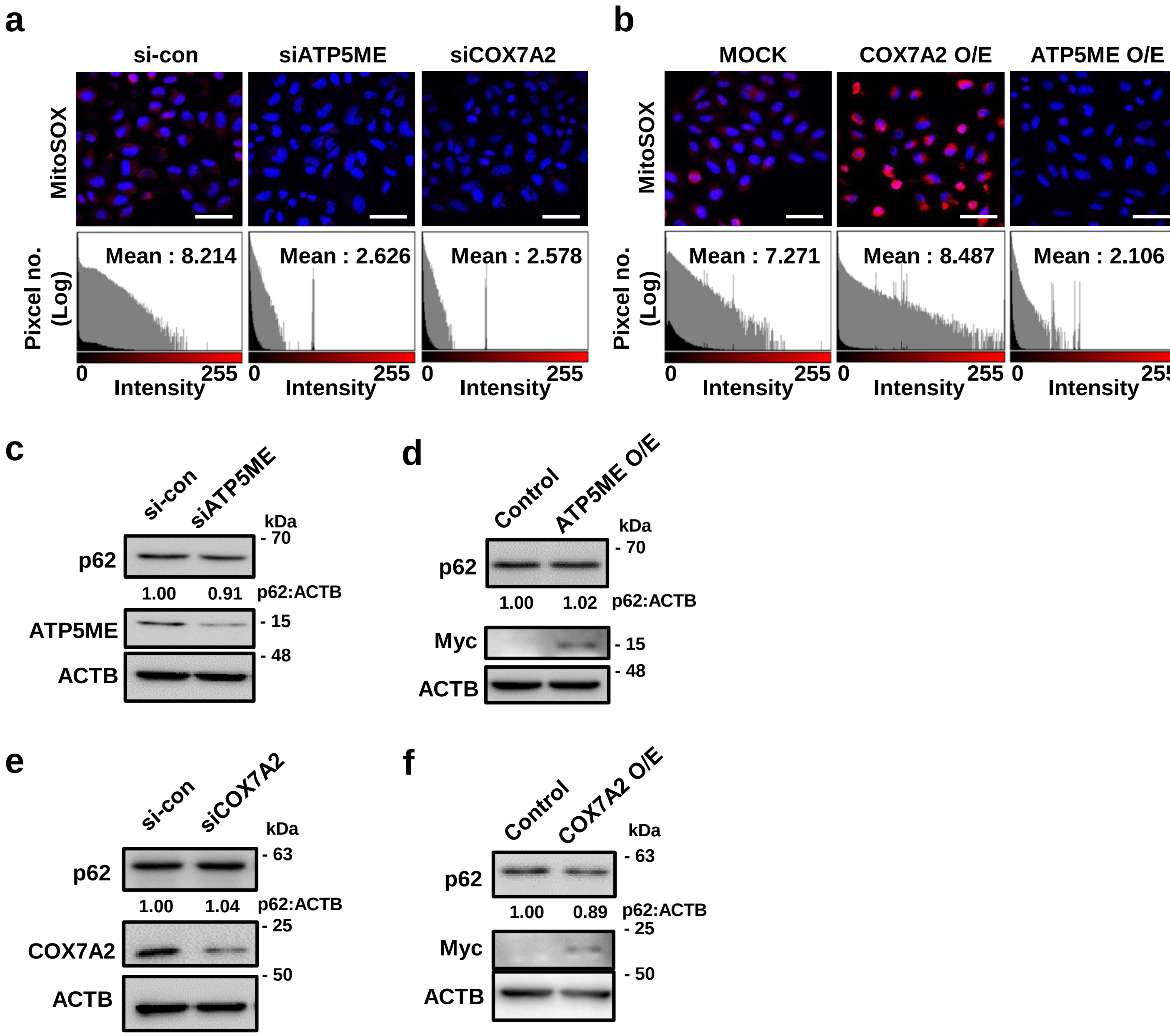

Supplementary Fig. 14 Other mitochondrial target candidates exhibited less impact on mtROS generation and autophagy. **a, b** HeLa cells were transfected with siRNA **a** or clone (MYC/DDK tagged) **b** for COX7A2 and ATP5I for 24 h. Cells were stained with mitoSOX and confocal microscopy performed (upper). Fluorescence intensity histogram generated using ImageJ 2 (lower). Scale bar, 50 μm. **c-f** HeLa cells were transfected with siRNA **c, e** or clone (MYC/DDK tagged) **d, f** for COX7A2 and ATP5I for 24 h. Cell extract was subjected to western blot analysis using antibodies against p62, COX7A2, ATP5I, and MYC. Numbers below p62 immunoblot bands indicates fold changes normalized to ACTB.

Supplementary Figure 15

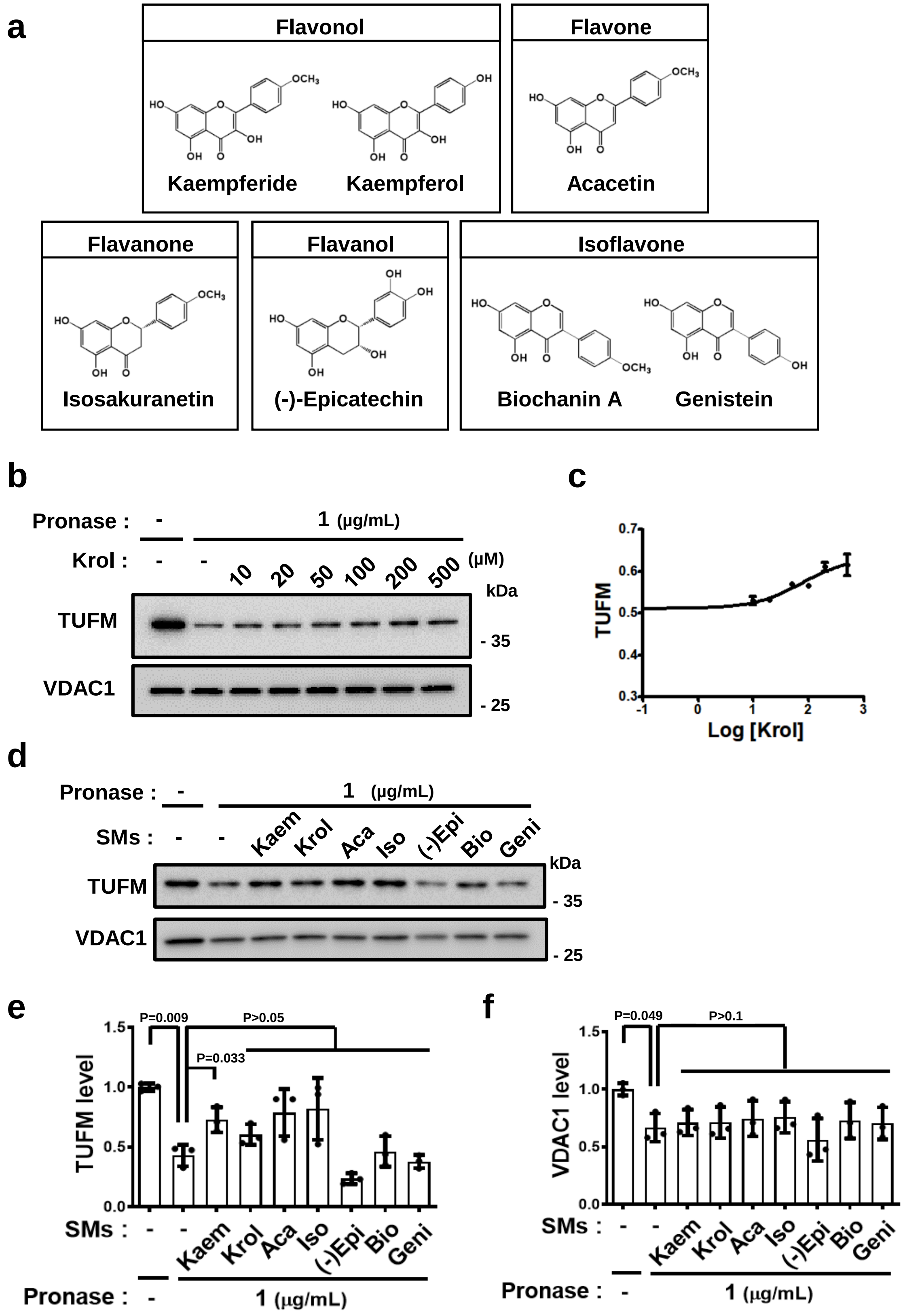

Supplementary Fig. 15 Structure-activity relationship of flavonoids against TUFM. **a** Structures of flavonoid small molecules (SMs) employed in this study. **b, c** 3T3-L1 cell lysate was treated with pronase for 10 minutes with or without kaempferol (Krol) pre-treatment in dose dependent manner (0, 10, 20, 50, 100, 200, and 500 μM). Western blot analysis was subjected using indicated antibodies. Representative images **b** and sigmoidal curve of band intensity **c**. **d-f** 3T3-L1 cell lysate was treated with pronase for 10 minutes with indicated chemicals pre-treatment (100 μM). Western blot analysis was subjected using indicated antibodies. Representative images **d** and intensity of TUFM **e** and VDAC1 **f** immunoblot bands. Graph shows mean ± SD from three independent experiments. Statistical significance was assessed by student's t-test. ***P<0.001; **P<0.01; *P<0.05.

Supplementary Figure 16

Fig. 1d

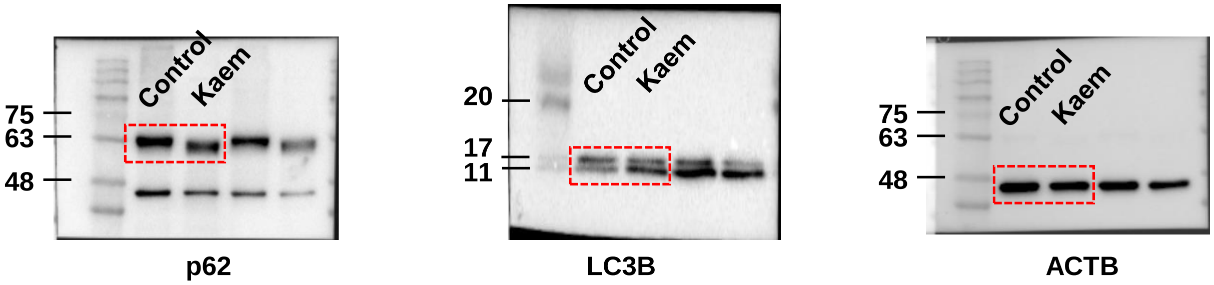

Fig. 1g

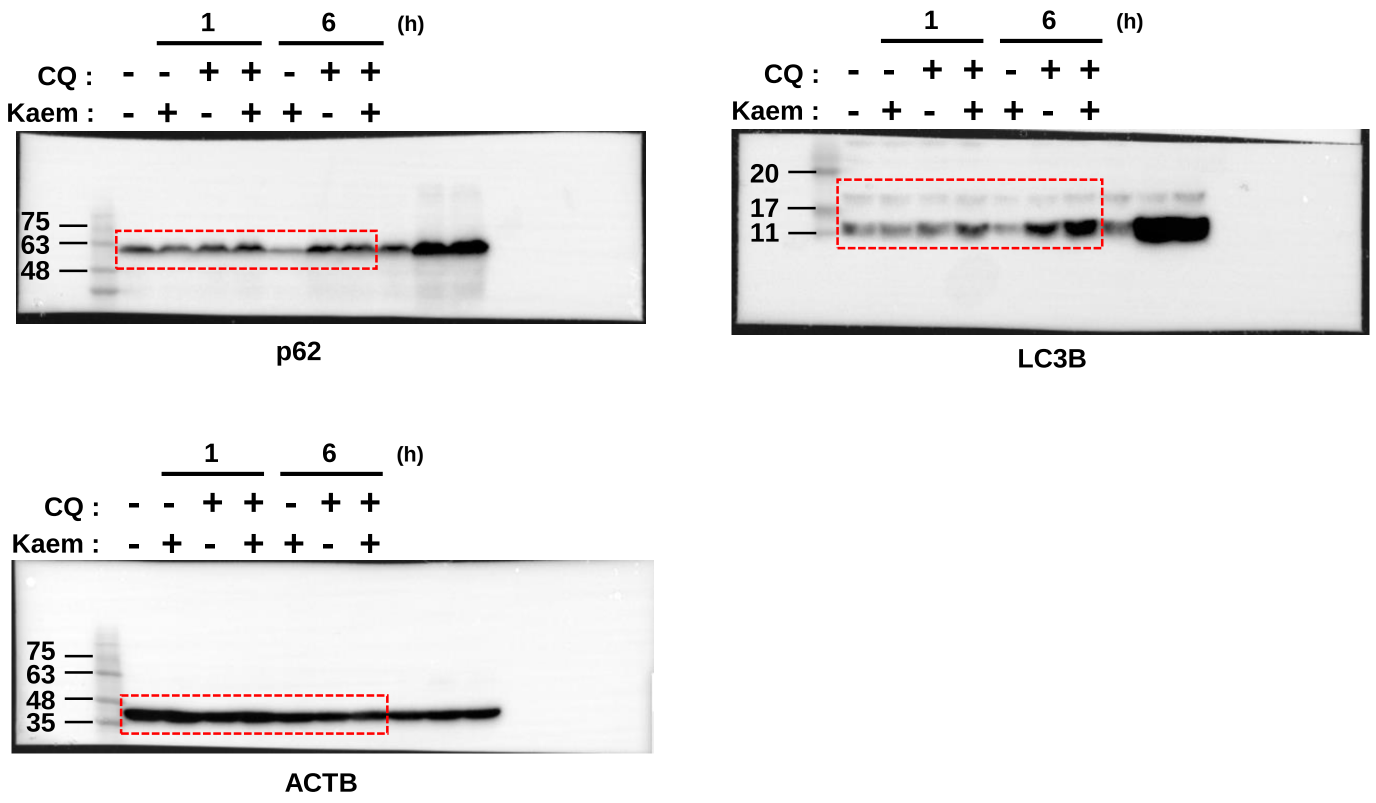

Supplementary Fig. 16 Original western blots shown in Figure 1. Each figures corresponds to the western blots in the indicated Figure number.

Supplementary Figure 17

Fig. 2a

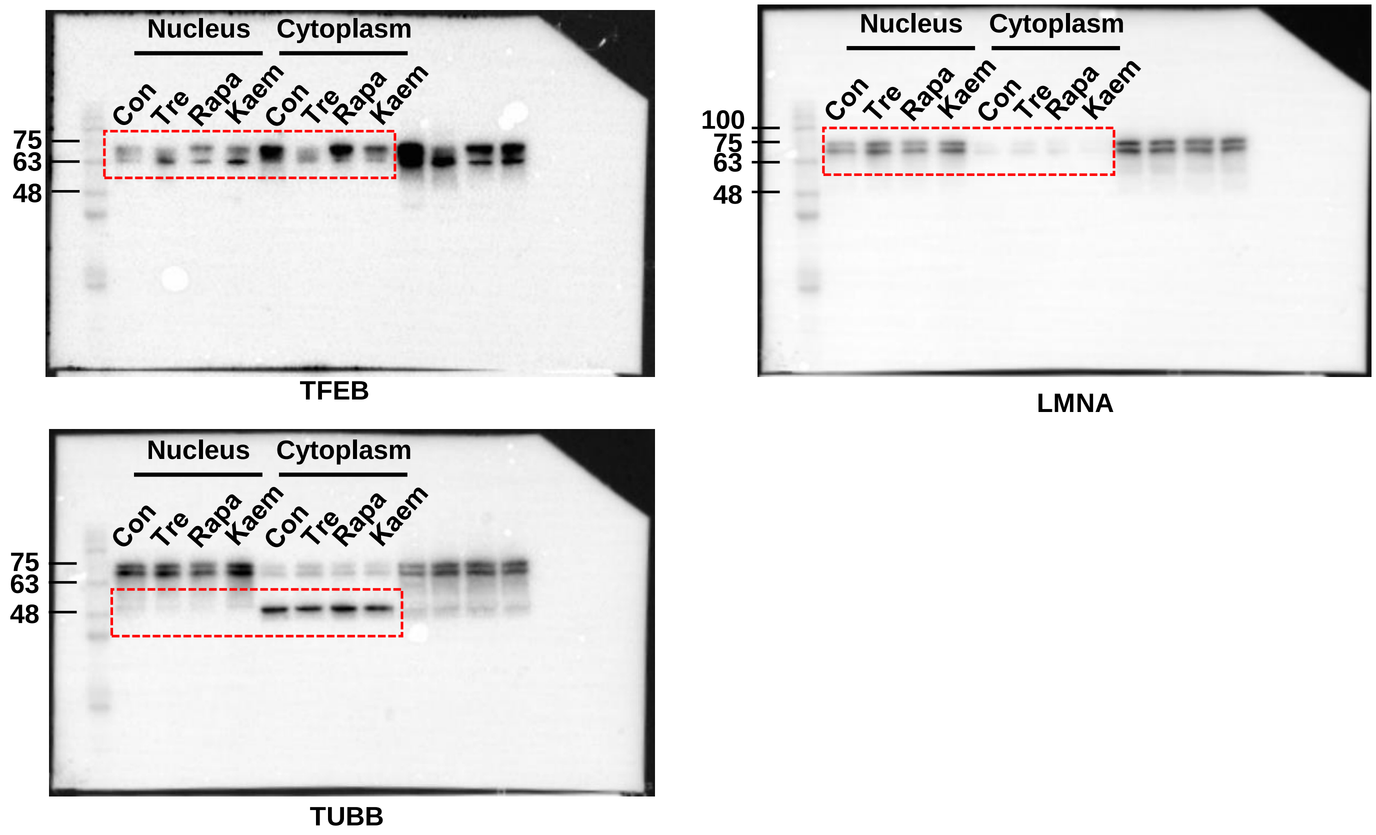

Fig. 2d

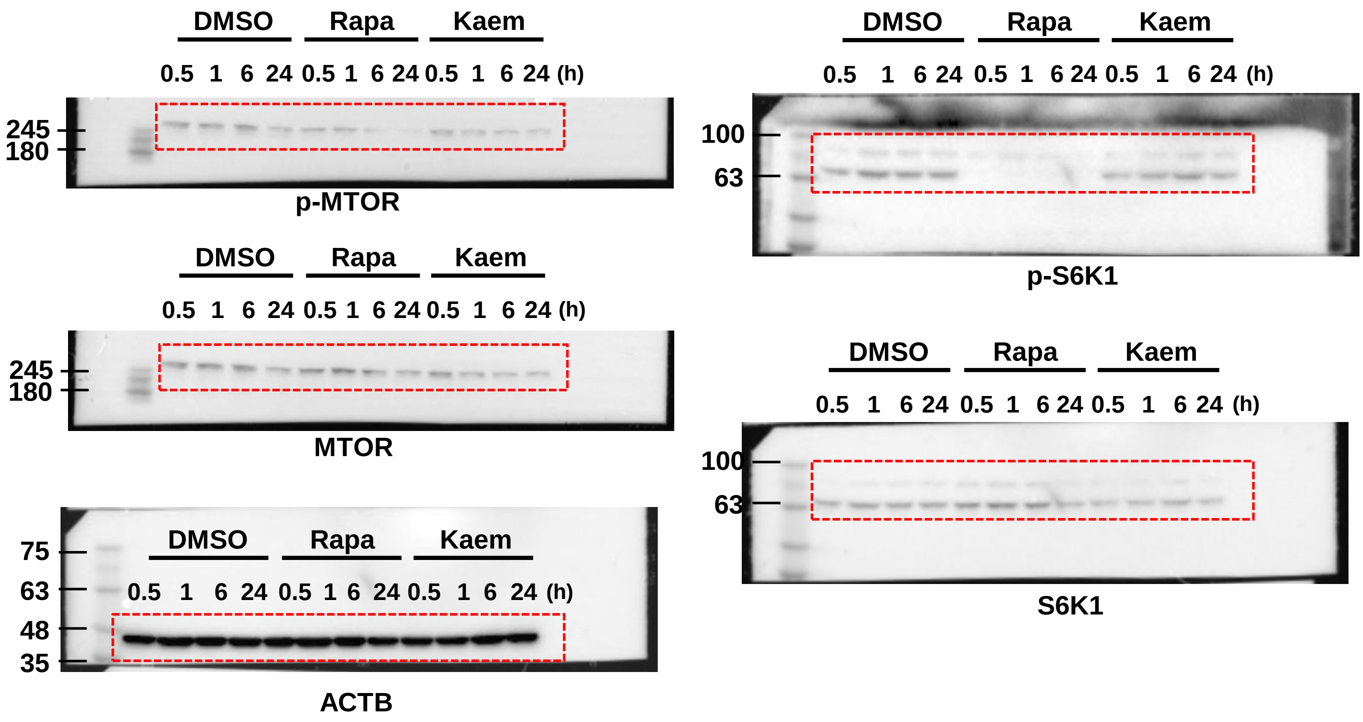

Supplementary Fig. 17 Original western blots shown in Figure 2. Each figures corresponds to the western blots in the indicated Figure number.

Supplementary Figure 18

Fig. 3c

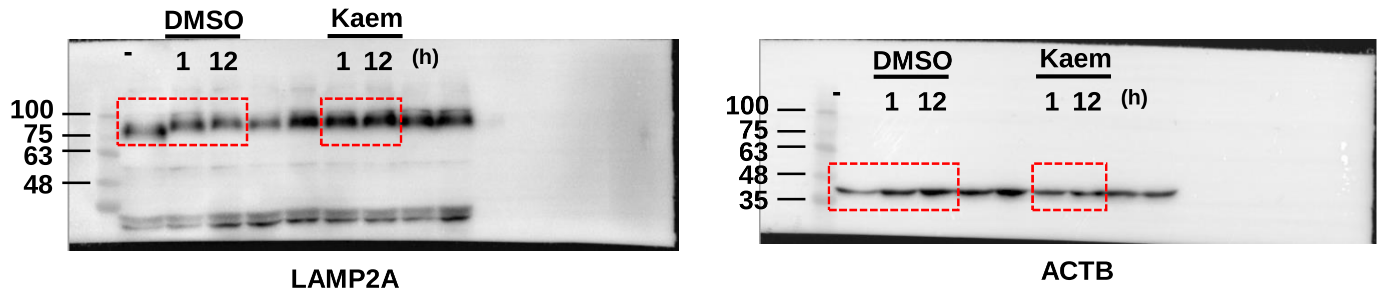

Fig. 3g

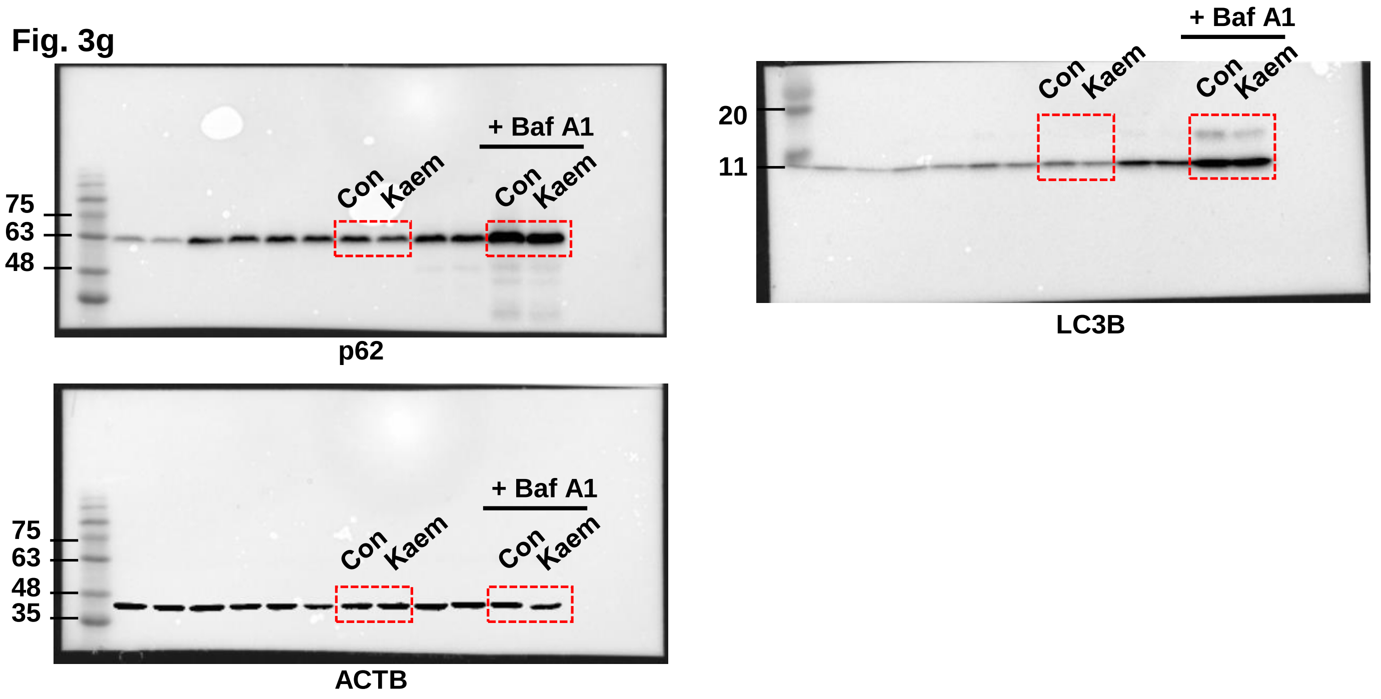

Fig. 4f

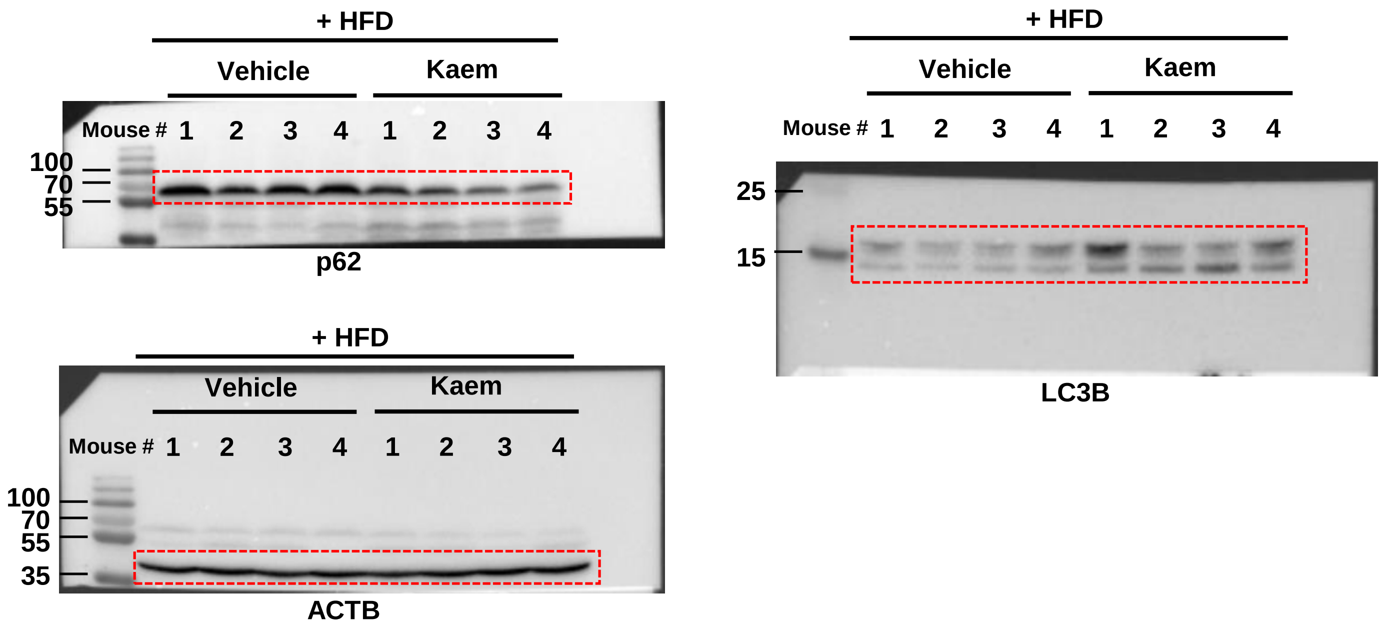

Supplementary Fig. 18 Original western blots shown in Figures 3-4. Each figures corresponds to the western blots in the indicated Figure number.

Supplementary Figure 19

Fig. 5d

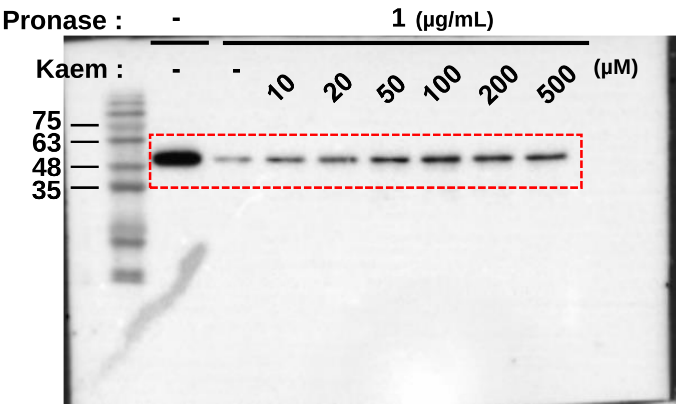

TUFM

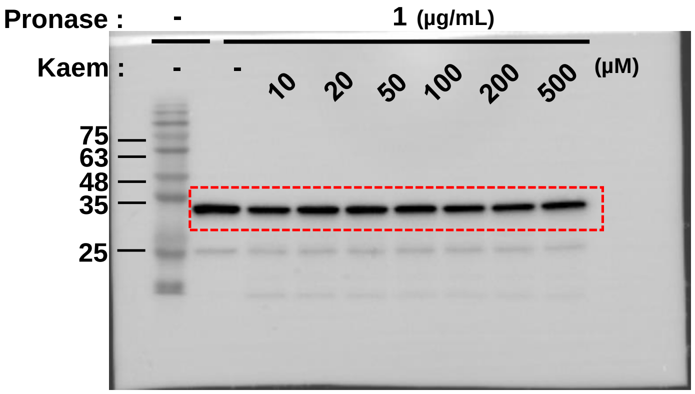

VDAC1

Fig. 6a

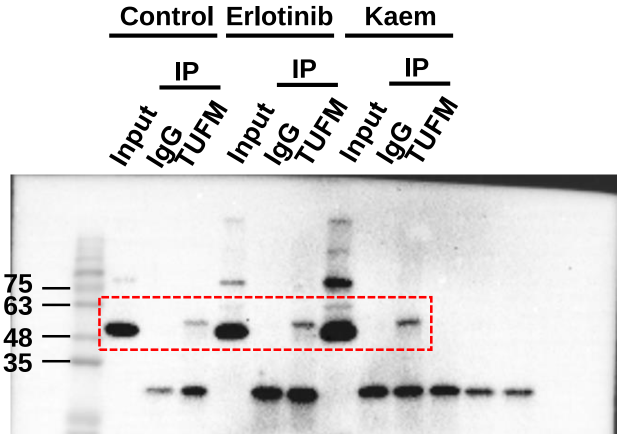

ATG12

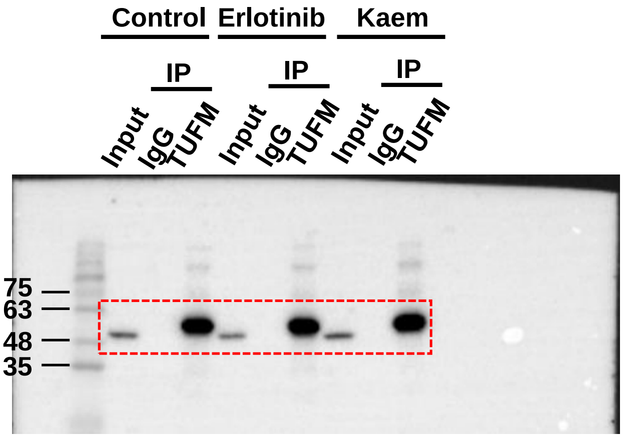

TUFM

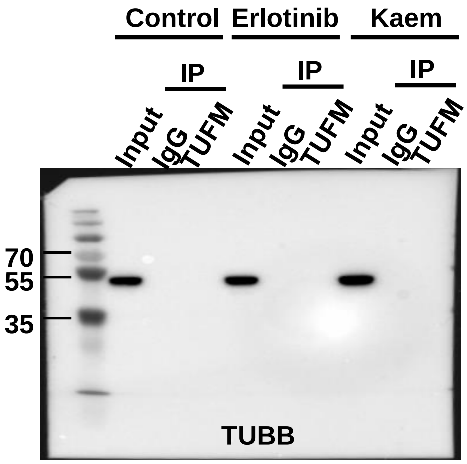

Supplementary Fig. 19 Original western blots shown in Figures 5-6. Each figures corresponds to the western blots in the indicated Figure number.

Supplementary Figure 20

Fig. 7a

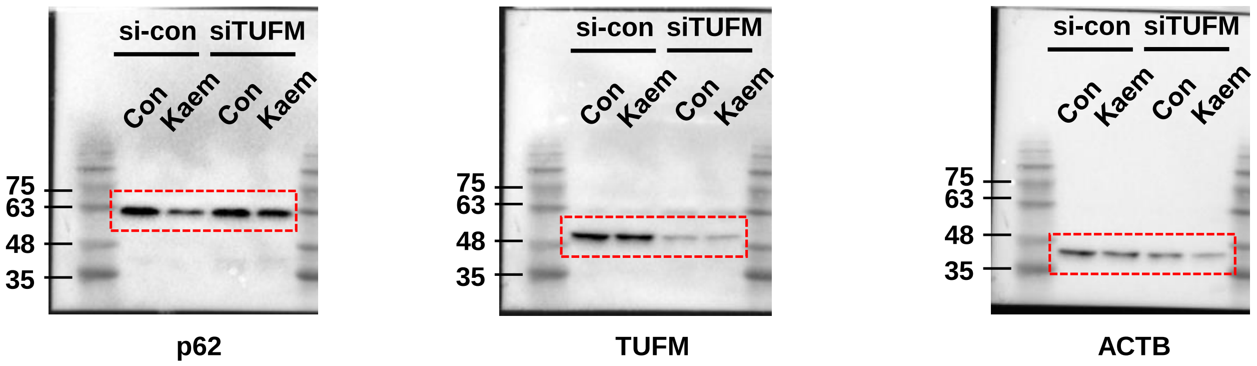

Fig. 7e

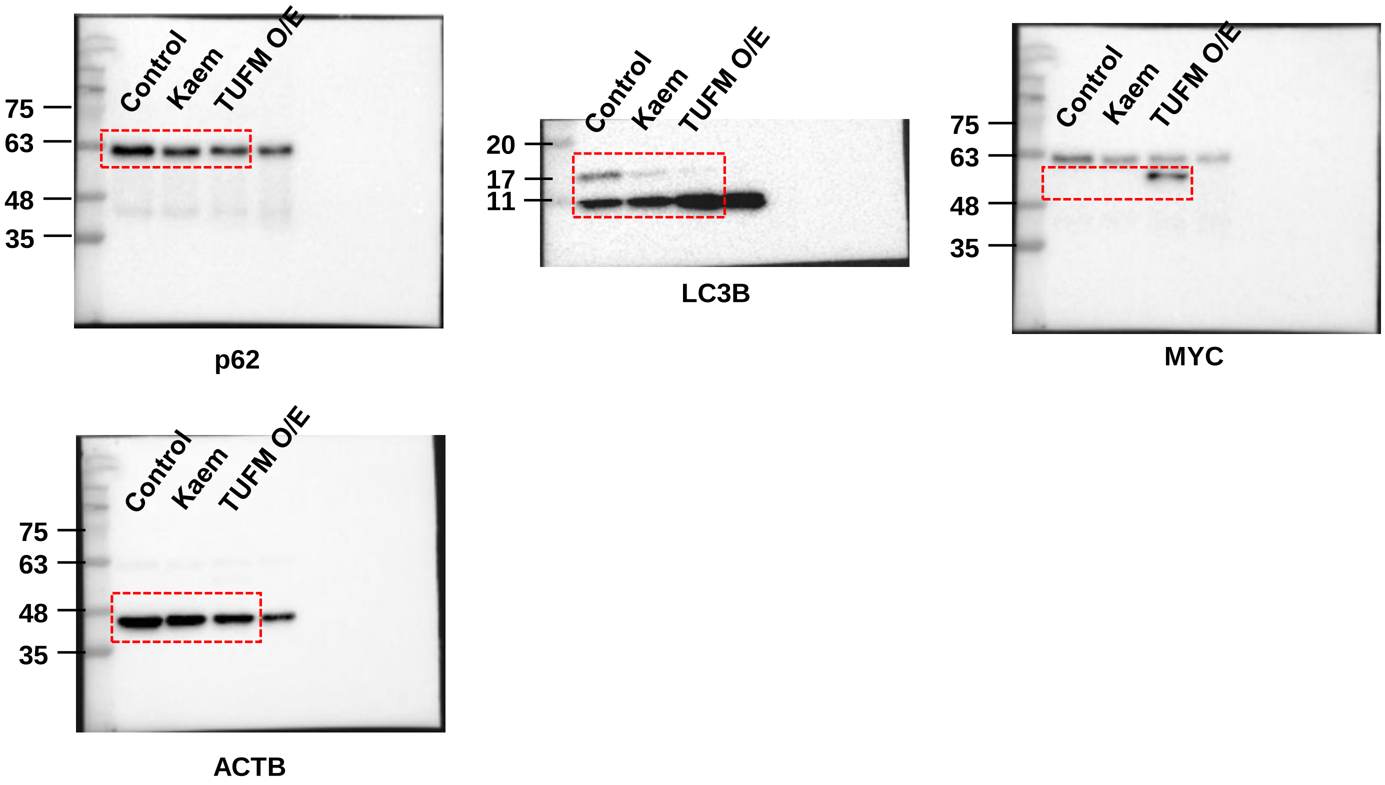

Supplementary Fig. 20 Original western blots shown in Figure 7. Each figures corresponds to the western blots in the indicated Figure number.

Supplementary Figure 21

Fig. 8a

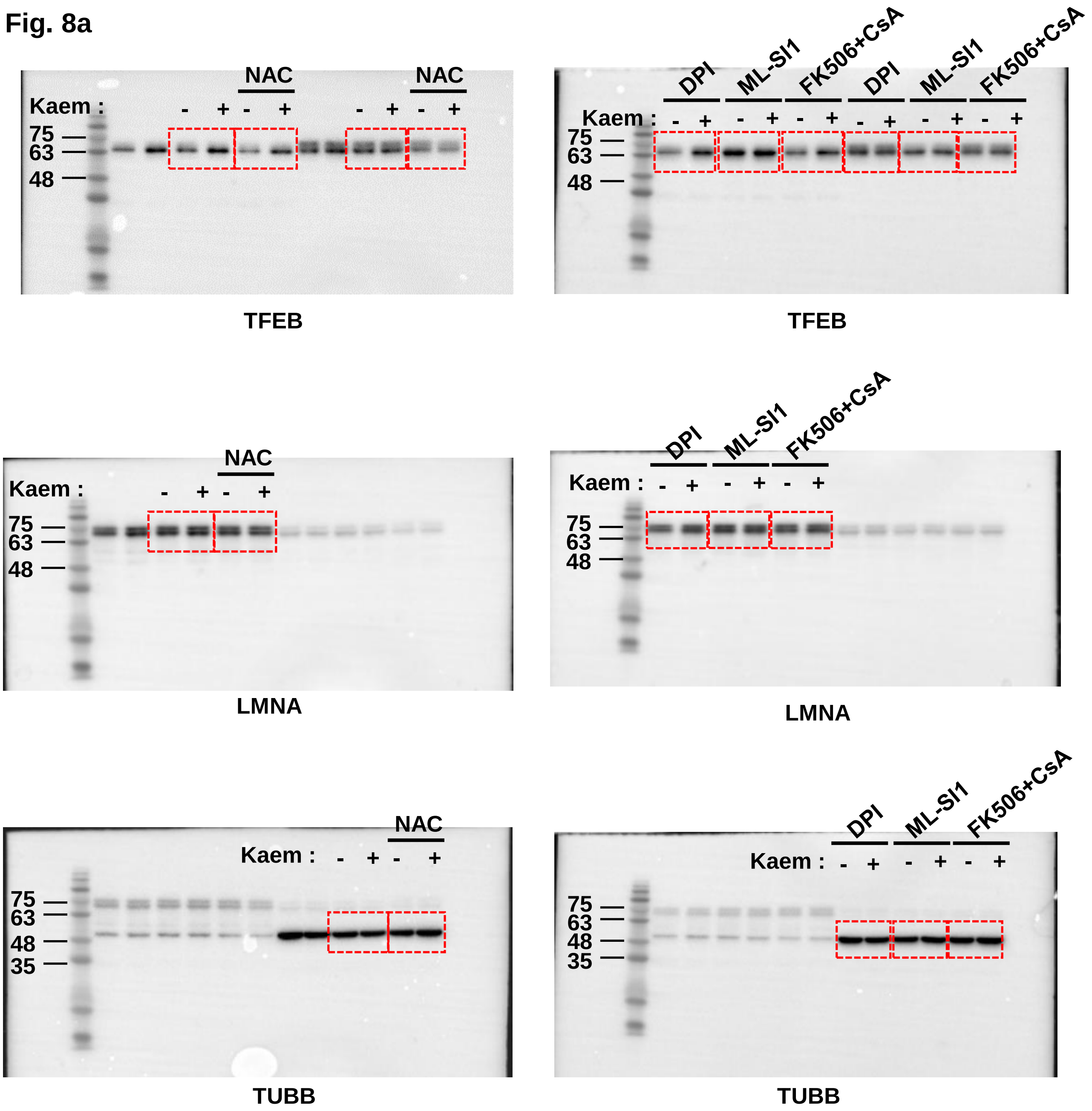

Fig. 8c

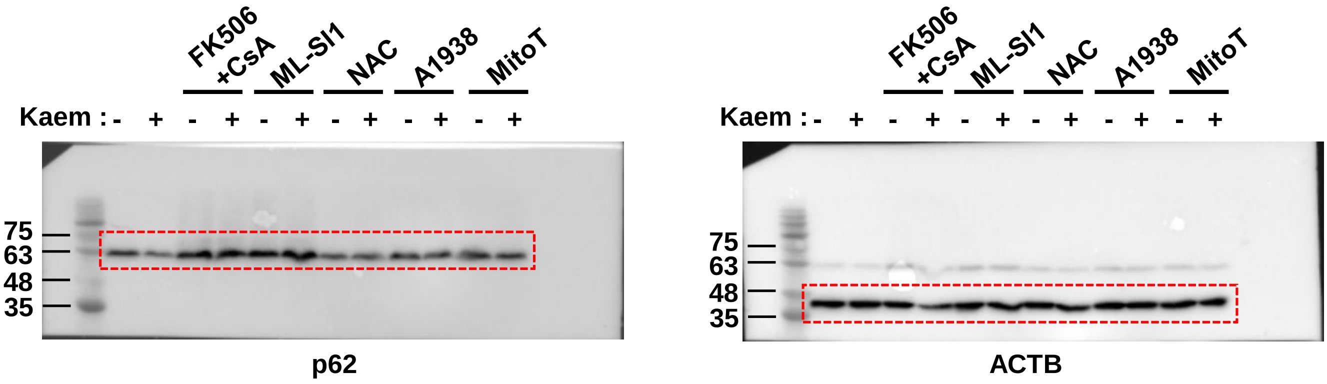

Supplementary Fig. 21 Original western blots shown in Figure 8. Each figures corresponds to the western blots in the indicated Figure number.

Supplementary Figure 22

Supplementary Fig. 3d

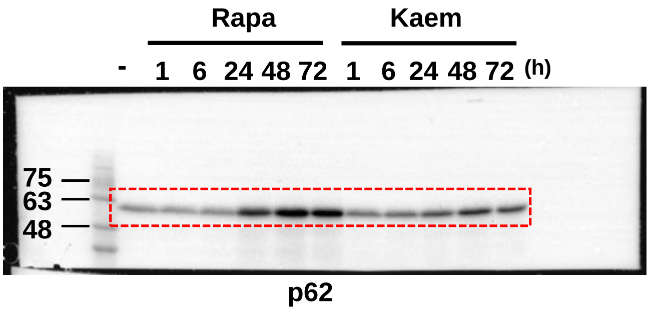

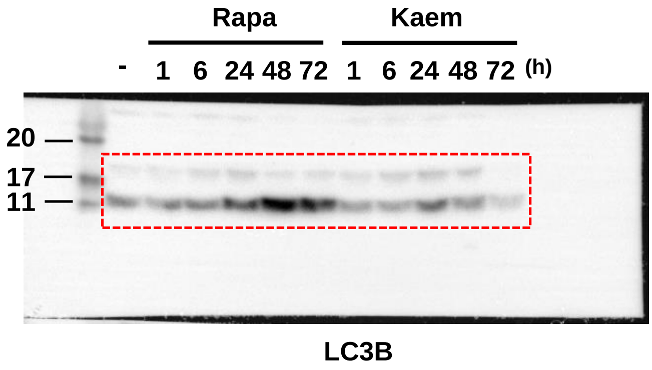

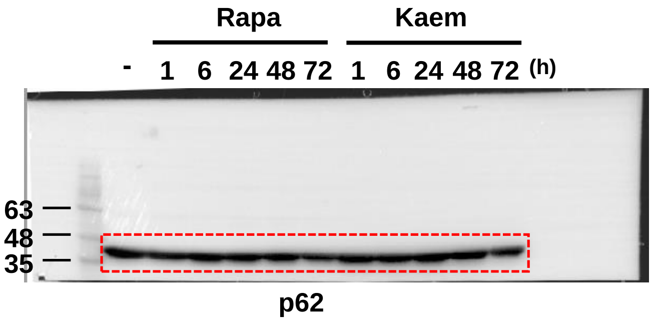

Supplementary Fig. 22 Original western blots shown in Supplementary Figure 3. Each figures corresponds to the western blots in the indicated Figure number.

Supplementary Figure 23

Supplementary Fig. 4a

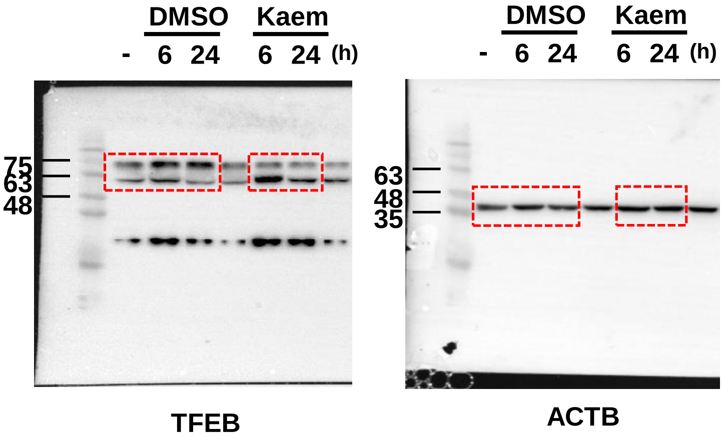

Supplementary Fig. 4b

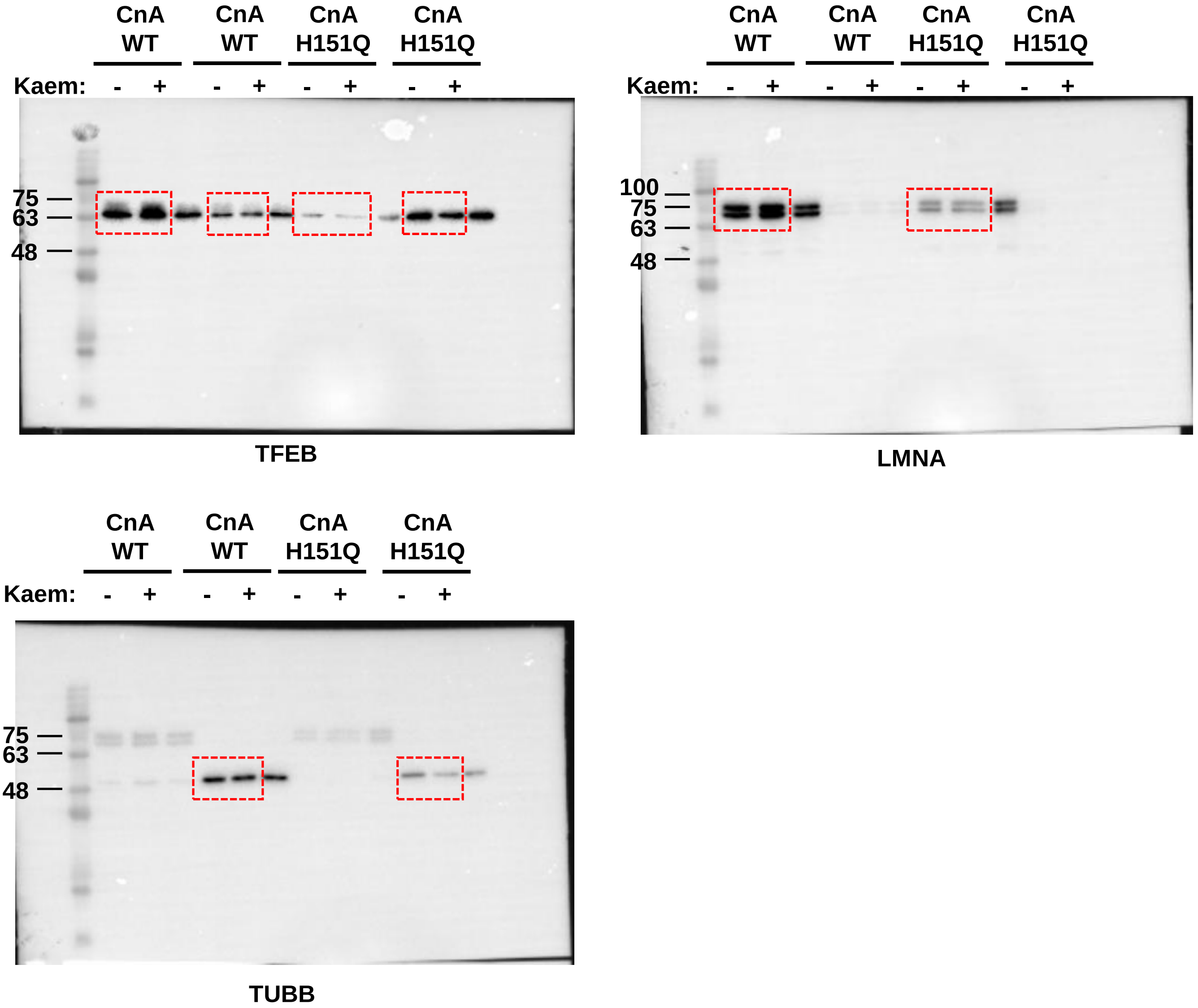

Supplementary Fig. 23 Original western blots shown in Supplementary Figure 4. Each figures corresponds to the western blots in the indicated Figure number.

Supplementary Figure 24

Supplementary Fig. 5a

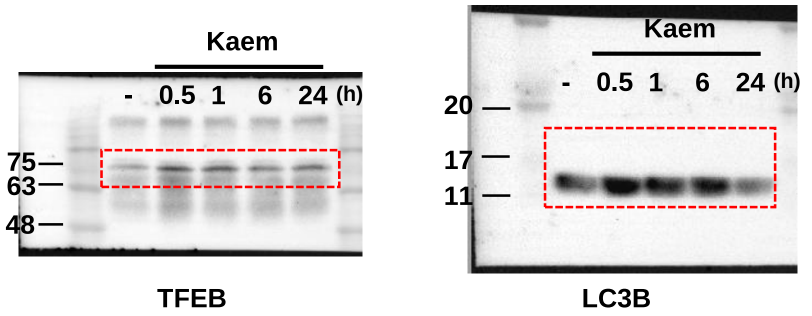

Supplementary Fig. 5b

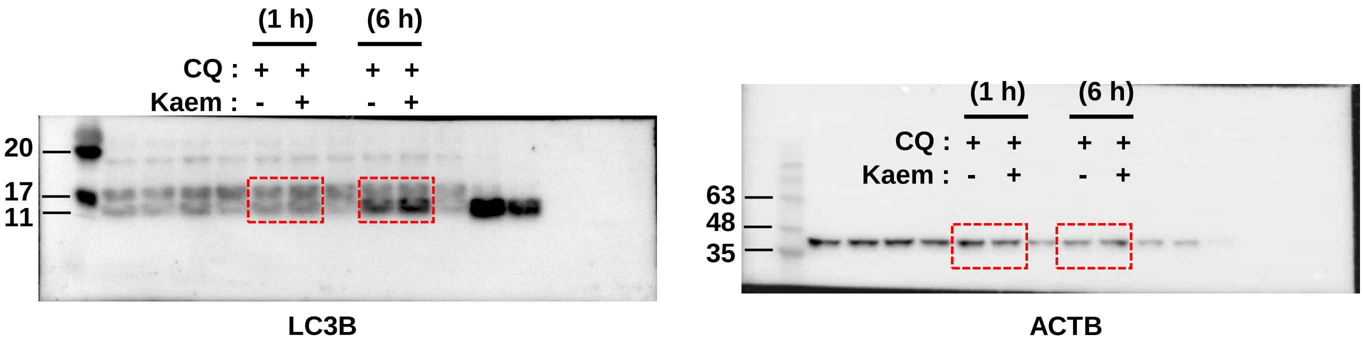

Supplementary Fig. 5c

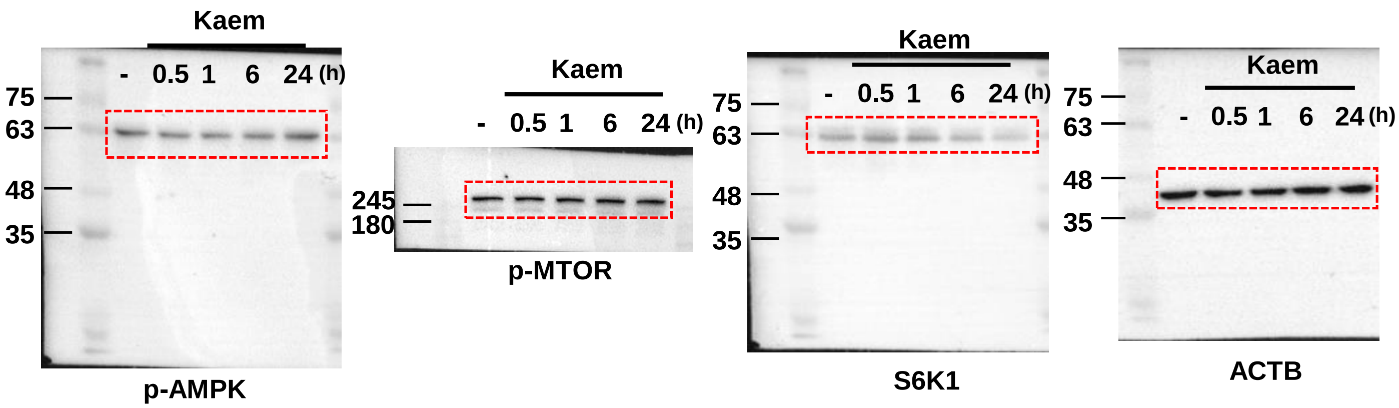

Supplementary Fig. 5h

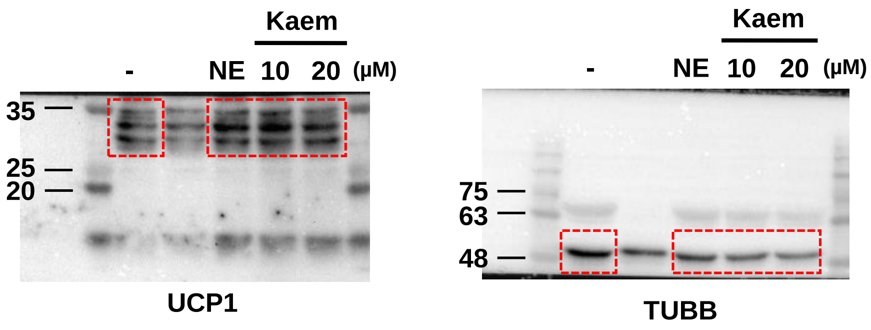

Supplementary Fig. 24 Original western blots shown in Supplementary Figure 5. Each figures corresponds to the western blots in the indicated Figure number.

Supplementary Figure 25

Supplementary Fig. 6c

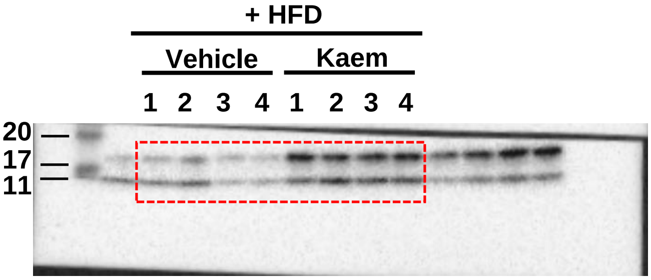

LC3B

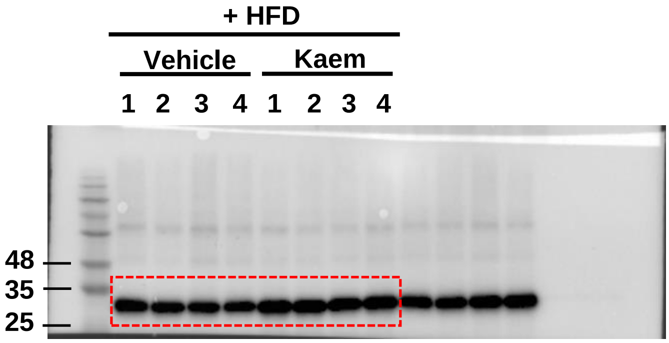

UCP1

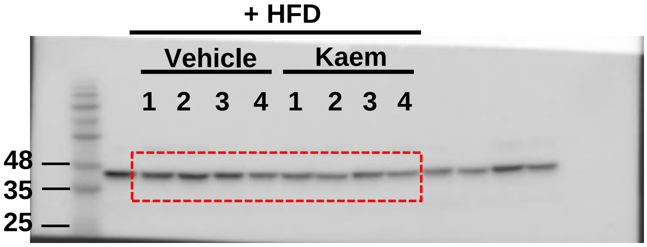

ACTB

Supplementary Fig. 6f

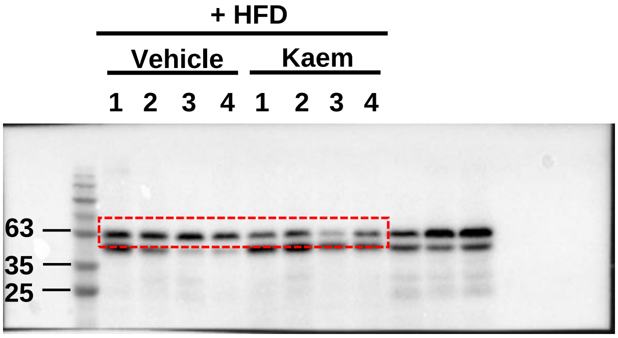

p62

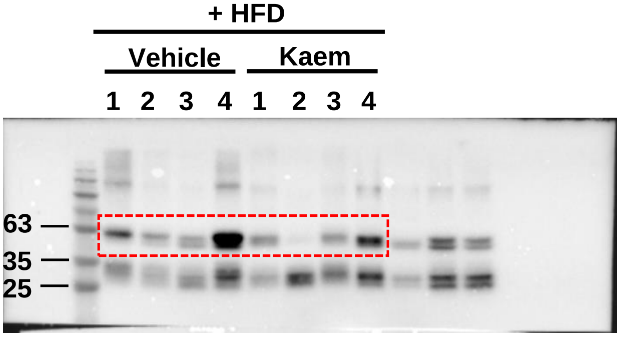

PLIN1

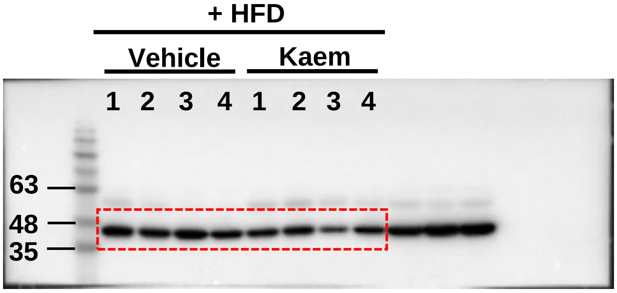

ACTB

Supplementary Fig. 25 Original western blots shown in Supplementary Figure 6. Each figures corresponds to the western blots in the indicated Figure number.

Supplementary Figure 26

Supplementary Fig. 9a

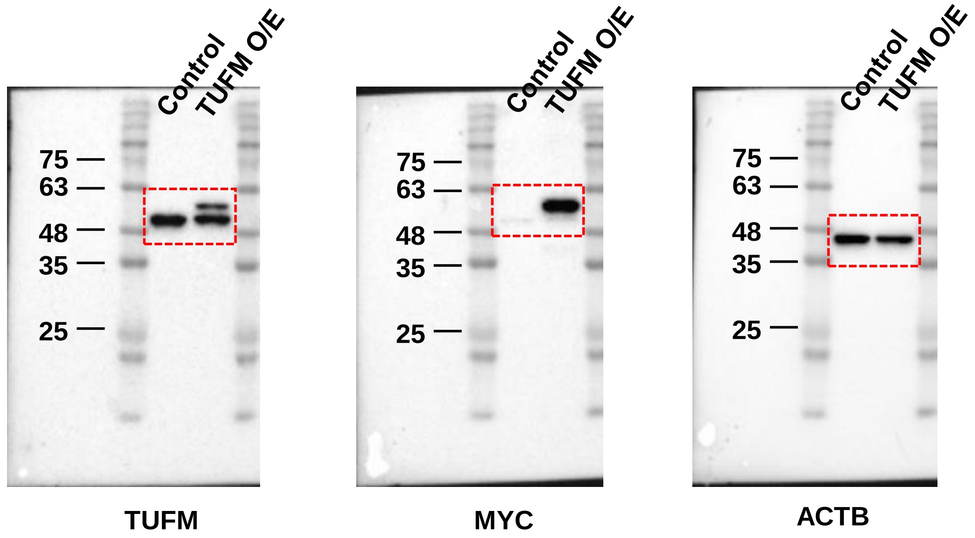

Supplementary Fig. 11b

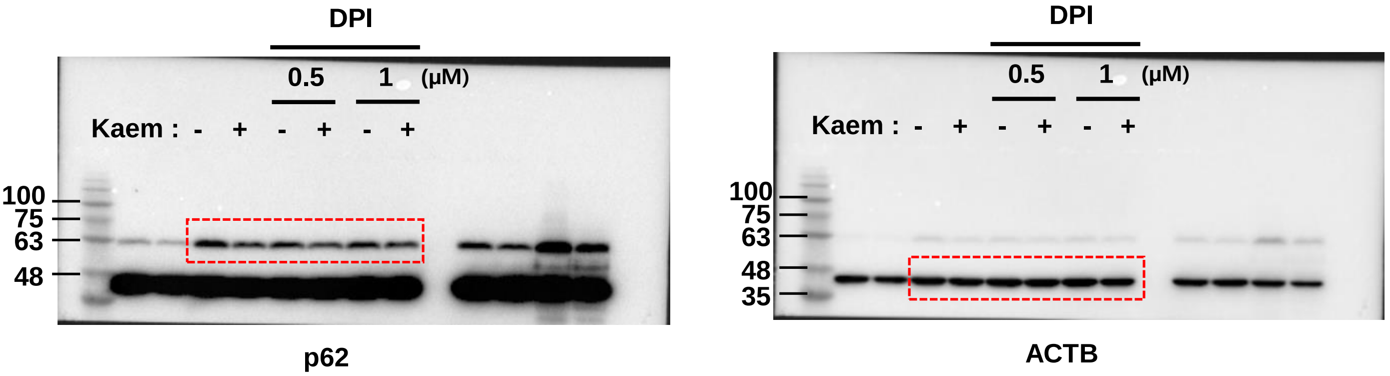

Supplementary Fig. 13

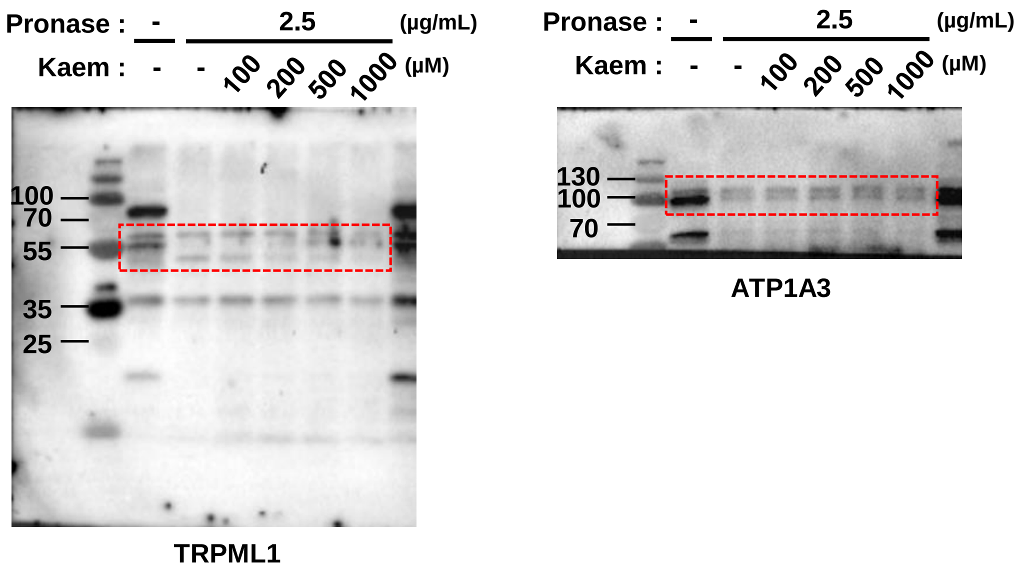

Supplementary Fig. 26 Original western blots shown in Supplementary Figures 9, 11, and 13. Each figures corresponds to the western blots in the indicated Figure number.

Supplementary Figure 27

Supplementary Fig. 14c

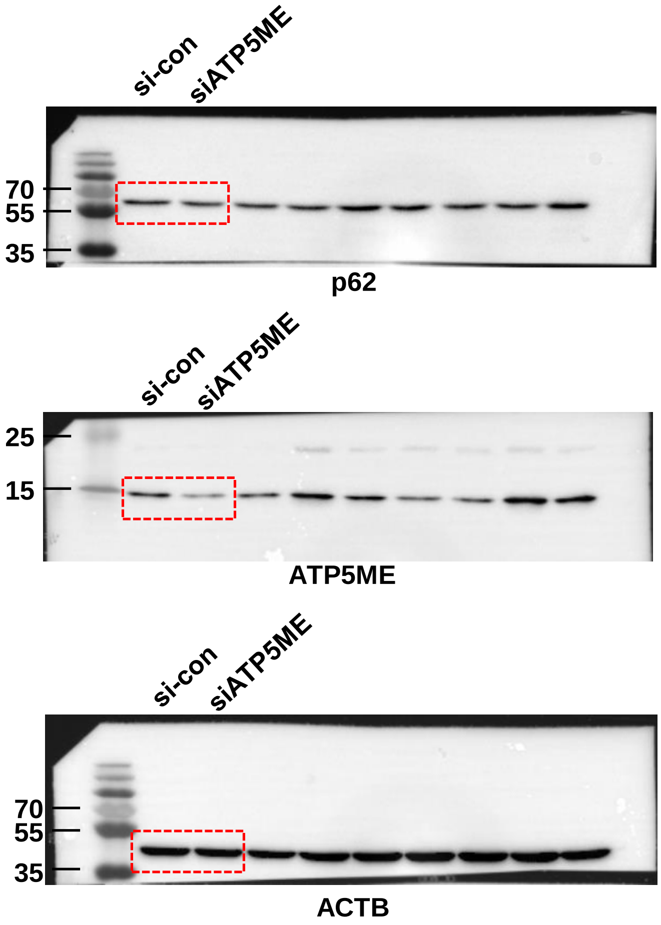

Supplementary Fig. 14e

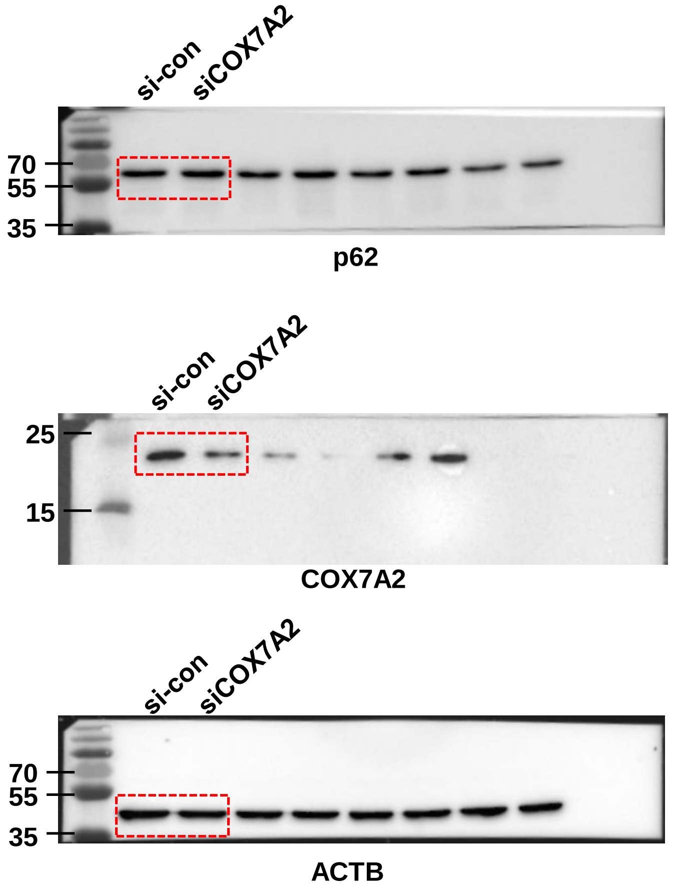

Supplementary Fig. 14d

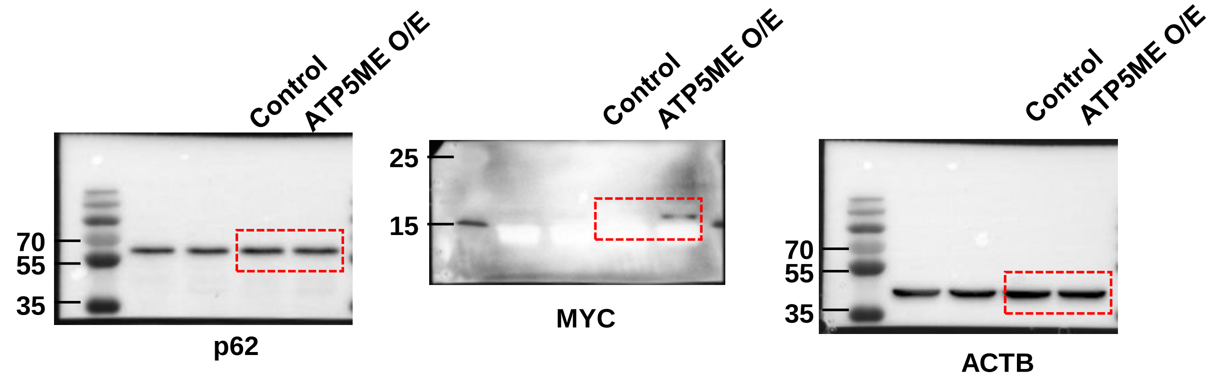

Supplementary Fig. 14f

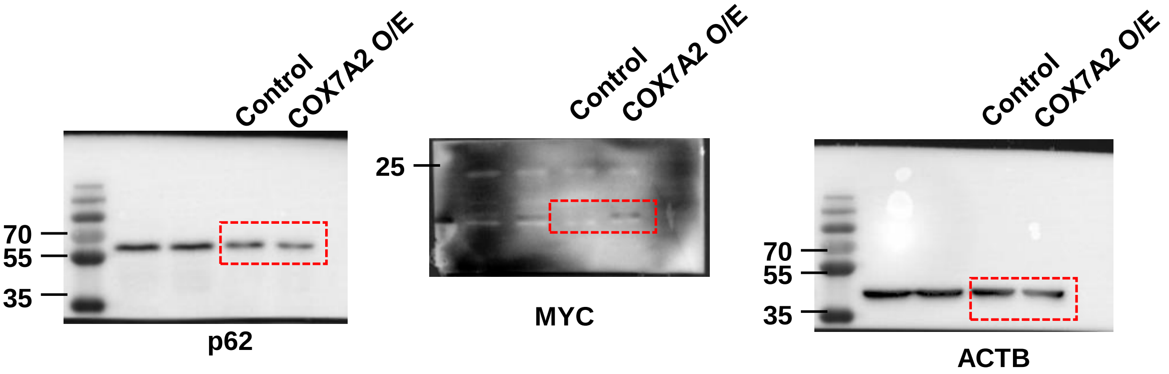

Supplementary Fig. 27 Original western blots shown in Supplementary Figure 14. Each figures corresponds to the western blots in the indicated Figure number.

Supplementary Figure 28

Supplementary Fig. 15b

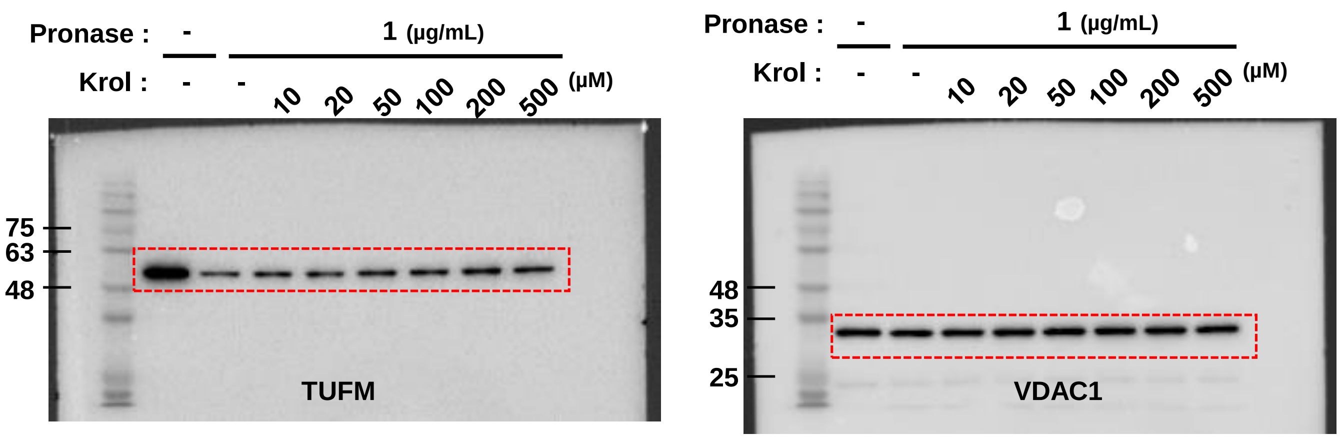

Supplementary Fig. 15d

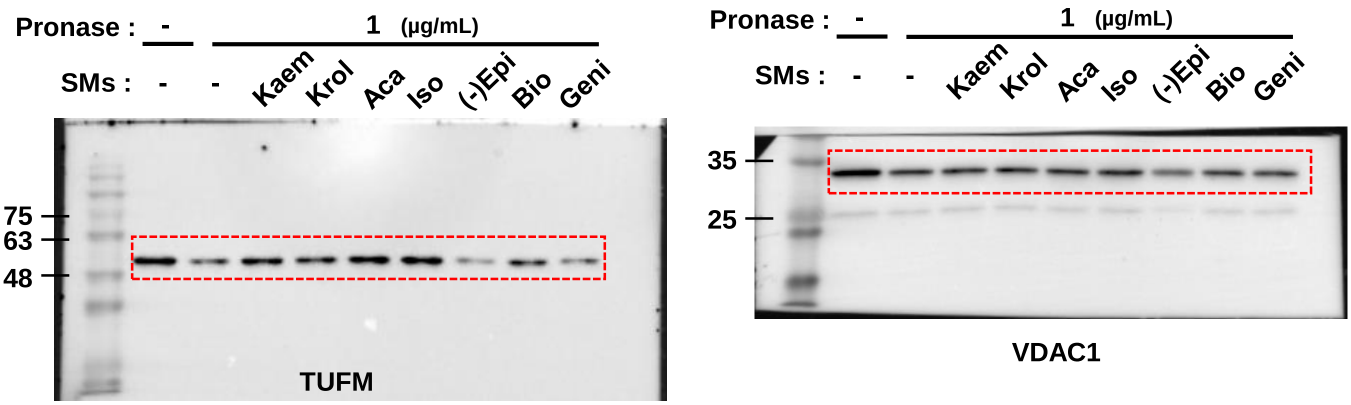

Supplementary Fig. 28 Original western blots shown in Supplementary Figure 15. Each figures corresponds to the western blots in the indicated Figure number.

Supplementary Table 1 List of antibodies

Target	Company	Catalog	Application
p62	BD biosciences	BD610833	1:2000 (WB)
p62	Abcam	ab56416	1:2000 (WB)
LC3B	Cell Signaling Technology	2775	1:2000 (WB)
LC3B	Abcam	ab48394	1:1000 (IF)
TFEB	Cell Signaling Technology	4240	1:1000 (WB)
LMNA	Santa Cruz	Sc-20681	1:2000 (WB)
p-MTOR	Cell Signaling Technology	2971	1:1000 (WB)
MTOR	Cell Signaling Technology	4517	1:1000 (WB)
p-S6K1	Cell Signaling Technology	9206	1:1000 (WB)
S6K1	Cell Signaling Technology	9202	1:1000 (WB)
LAMP2A	Abcam	ab18528	1:1000 (WB)
TUFM	Atlas Antibodies	AMb90964	1:1000 (WB)
VDAC1	Abcam	ab154856	1:2000 (WB)
ATG12	Cell Signaling Technology	4180	1:1000 (WB)
ACTB	Abcam	ab6276	1:10000 (WB)
TUBB	Abcam	ab6046	1:5000 (WB)
UCP1	Abcam	ab10983	1:1000 (WB)
PLIN1	Cell Signaling Technology	9349	1:1000 (WB)
ATG7	Cell Signaling Technology	8558	1:1000 (WB)
RAB9	Cell Signaling Technology	5133	1:1000 (WB)
RAB5	Cell Signaling Technology	3547	1:1000 (WB)
MYC	MBL	M192-3	1:2000 (WB)
TRPML1	Novus Biologicals	NB110-82375	1:2000 (WB)
ATP1A3	Abcam	ab2826	1:1000 (WB)
ATP5ME	Abcam	ab122241	1:2000 (WB)
COX7A2	Invitrogen	PA5-99611	1:1000 (WB)
