# Supplementary material for: Activation of mitochondrial TUFM ameliorates metabolic dysregulation through coordinating autophagy induction
Source: Commun Biol. 2021 Jan 4;4:1. doi: 10.1038/s42003-020-01566-0 (PMC7782552; doi:10.1038/s42003-020-01566-0)
Supplement: Supplementary file 3 — Description of Additional Supplementary Files [file 42003_2020_1566_MOESM3_ESM.docx]

**Description of Additional Supplementary Items**

**File name:** Supplementary Data 1

**Description:** This file contains all source data underlying the screening results. This includes results shown in Fig. S1b.

**File name:** Supplementary Data 2

**Description:** This file contains all source data underlying the *in vivo* experiment results for body weight and blood glucose levels in Excel format. This includes results shown in Fig. 4a-d.

**File name:** Supplementary Data 3

**Description:** This file contains all source data underlying the DARTS-LC-MS/MS results in Excel format. This includes results shown in Fig. 5a and Fig. S7.

**File name:** Supplementary Data 4

**Description:** This file contains all source data underlying the peptide sequence of TUFM in LC-MS/MS results in Excel format. This includes results shown in Fig. 5g-h.

**File name:** Supplementary Data 5

**Description:** This file contains all source data underlying the *in silico* docking analysis in Excel format. This includes results shown in Fig. 5i.

**File name:** Supplementary Data 6

**Description:** This file contains all source data underlying the LC-MS/MS data for mitochondrial proteome analysis in Excel format. This includes results shown in Fig. 8f and Fig. S12.

**File name:** Supplementary Data 7

**Description:** This file contains all source data underlying the LC-MS/MS data for cytoplasmic proteome analysis in Excel format. This includes results shown in Fig. S5e.

**File name:** Supplementary Data 8

**Description:** This file contains all source data for the graphs in the main figures.
