# Supplementary material for: Activation of mitochondrial TUFM ameliorates metabolic dysregulation through coordinating autophagy induction
Source: Commun Biol. 2021 Jan 4;4:1. doi: 10.1038/s42003-020-01566-0 (PMC7782552; doi:10.1038/s42003-020-01566-0)
Supplement: Supplementary file 12 — Reporting Summary [file 42003_2020_1566_MOESM12_ESM.pdf]

nature research

Corresponding author(s): Ho Jeong KWON

Last updated by author(s): Nov 25, 2020

Reporting Summary

Nature Research wishes to improve the reproducibility of the work that we publish. This form provides structure for consistency and transparency in reporting. For further information on Nature Research policies, see our Editorial Policies and the Editorial Policy Checklist.

Statistics

For all statistical analyses, confirm that the following items are present in the figure legend, table legend, main text, or Methods section.

n/a	Confirmed	
☐	☒	The exact sample size (n) for each experimental group/condition, given as a discrete number and unit of measurement
☐	☒	A statement on whether measurements were taken from distinct samples or whether the same sample was measured repeatedly
☐	☒	The statistical test(s) used AND whether they are one- or two-sided<br>*Only common tests should be described solely by name; describe more complex techniques in the Methods section.*
☒	☐	A description of all covariates tested
☒	☐	A description of any assumptions or corrections, such as tests of normality and adjustment for multiple comparisons
☐	☒	A full description of the statistical parameters including central tendency (e.g. means) or other basic estimates (e.g. regression coefficient) AND variation (e.g. standard deviation) or associated estimates of uncertainty (e.g. confidence intervals)
☒	☐	For null hypothesis testing, the test statistic (e.g. F, t, r) with confidence intervals, effect sizes, degrees of freedom and P value noted<br>*Give P values as exact values whenever suitable.*
☒	☐	For Bayesian analysis, information on the choice of priors and Markov chain Monte Carlo settings
☒	☐	For hierarchical and complex designs, identification of the appropriate level for tests and full reporting of outcomes
☒	☐	Estimates of effect sizes (e.g. Cohen's d, Pearson's r), indicating how they were calculated

Our web collection on statistics for biologists contains articles on many of the points above.

Software and code

Policy information about availability of computer code

Data collection	no software was used to collect data.
Data analysis	'Chemdraw Ultra7.0' for drawing chemical structure; 'Image Lab' for acquisition of gel and immunoblot images; 'ZEN black' for fluorescent images processing; 'imageJ2' for quantification of immunoblot and fluorescence images; 'Discovery Studio' for in silico docking analysis and chemical properties analysis; 'GraphPad Prism 5 or 8' for drawing graph and statistical analysis

For manuscripts utilizing custom algorithms or software that are central to the research but not yet described in published literature, software must be made available to editors and reviewers. We strongly encourage code deposition in a community repository (e.g. GitHub). See the Nature Research guidelines for submitting code & software for further information.

Data

Policy information about availability of data

All manuscripts must include a data availability statement. This statement should provide the following information, where applicable:
- Accession codes, unique identifiers, or web links for publicly available datasets
- A list of figures that have associated raw data
- A description of any restrictions on data availability

The data that support the findings of this study are included in the paper and its supplementary information files or available from the corresponding author upon request.

Field-specific reporting

Please select the one below that is the best fit for your research. If you are not sure, read the appropriate sections before making your selection.

- [x] Life sciences
- [ ] Behavioural & social sciences
- [ ] Ecological, evolutionary & environmental sciences

For a reference copy of the document with all sections, see nature.com/documents/nr-reporting-summary-flat.pdf

Life sciences study design

All studies must disclose on these points even when the disclosure is negative.

Sample size	Sample sizes were chosen based on those published in previous studies.
Data exclusions	No data were excluded from the manuscript.
Replication	Quantitative data were obtained from at least three biological independent experiments unless differently specified.
Randomization	Individual animals were randomly chosen and allocated into experimental groups for the phenotype analyses.
Blinding	Data were batch-analyzed and/or quantified using softwares so blinding to group allocation was not necessarily applicable.

Reporting for specific materials, systems and methods

We require information from authors about some types of materials, experimental systems and methods used in many studies. Here, indicate whether each material, system or method listed is relevant to your study. If you are not sure if a list item applies to your research, read the appropriate section before selecting a response.

Materials & experimental systems

n/a	Involved in the study	
☐	☒	Antibodies
☐	☒	Eukaryotic cell lines
☒	☐	Palaeontology and archaeology
☐	☒	Animals and other organisms
☒	☐	Human research participants
☒	☐	Clinical data
☒	☐	Dual use research of concern

Methods

n/a	Involved in the study	
☒	☐	ChIP-seq
☒	☐	Flow cytometry
☒	☐	MRI-based neuroimaging

Antibodies

Antibodies used

Western blot:
Primary antibodies: p62 (610833, BD Biosciences or 56416, Abcam), LC3B (2775, Cell Signaling Technology or 51520, Abcam), TFEB (4240, Cell Signaling Technology or 32361, Cell Signaling Technology), Lamin A/C (20681, Santa Cruz Biotechnology), p-MTOR(S2448) (2971, Cell Signaling Technology), MTOR (4517, Cell Signaling Technology), p-S6K1(T389) (9206, Cell Signaling Technology), S6K1 (9202, Cell Signaling Technology), LAMP2A (18528, Abcam), TUFM (AMAb90964, Atlas Antibodies), COX7A2 (PA5-99611, Invitrogen), ATP5I (ab122241, Abcam), VDAC1 (154856, Abcam), ATG12 (4180, Cell Signaling Technology), MYC (M192-3, MBL), p-AMPK(T172) (2535, Cell Signaling Technology), AMPK (2532, Cell Signaling Technology), UCP1 (10983, Abcam), PLIN1 (3526, Abcam), TRPML1 (NB110-82375, Novus Biologicals), ATP1A3 (2826, Abcam), TUBB (6046, Abcam), ACTB (6276 Abcam or 8227 Abcam)
Secondary antibodies: Anti-mouse (SA001-500, GenDepot), Anti-rabbit (SA002-500, GenDepot)

Cell culture immunofluorescent staining:
Primary antibodies: TFEB (220695, Abcam), HA (18181, Abcam), LC3 (48394, Abcam), TUFM (AMAb90964, Atlas Antibodies), ATG12 (4180, Cell Signaling Technology), MYC (M192-3, MBL)
Secondary antibodies: Alexa 594 anti-mouse (11005, invitrogen), Alexa 594 anti-rabbit (11012, invitrogen), Alexa 488 anti-mouse (32723, invitrogen), Alexa 488 anti-rabbit (11008, invitrogen)

In vivo liver tissue immunofluorescent staining:
Primary antibody: LC3 (48394, Abcam)
Secondary antibody: Alexa 594 anti-rabbit (11012, invitrogen)

Validation

All antibodies were validated by manufacturers as noted on data sheets. The antibodies are commonly used, used in independent publications and catalogs numbers can be used to look up additional validation experiments.

Eukaryotic cell lines

Policy information about cell lines

Cell line source(s)	HeLa (ATCC® CCL-2), HepG2 (ATCC® HB-8065) were obtained from the American Type Culture Collection (ATCC). Huh7, 3T3-L1 were obtained from Korean Cell Line Bank.
Authentication	Cell authentication was based on morphological criteria.
Mycoplasma contamination	Mycoplasma contamination of cell culture was periodically checked, and cells were found negative for the presence of mycoplasma species.
Commonly misidentified lines (See ICLAC register)	No commonly misidentified cell lines were used.

Animals and other organisms

Policy information about studies involving animals; ARRIVE guidelines recommended for reporting animal research

Laboratory animals	Species: Mus musculus, Strain: C57BL/6j, Sex: male, Age: 4-20 weeks, Raon-bio
Wild animals	No wild animals was used in this study.
Field-collected samples	This study does not involve samples collected from the field.
Ethics oversight	Animal studies were approved and performed in accordance with the guidelines of the Institutional Animal Care and Use Committee of Yonsei University after approval (IACUC-A-201806-741-02) and conformed to the Guide for Care and Use of Laboratory Animals published by the US National Institutes of Health (The National Academies Press, 8th Edition, 2011).

Note that full information on the approval of the study protocol must also be provided in the manuscript.
